# Stochastic Boolean model of normal and aberrant cell cycles in budding yeast

**DOI:** 10.1038/s41540-024-00452-3

**Published:** 2024-10-18

**Authors:** Kittisak Taoma, John J. Tyson, Teeraphan Laomettachit, Pavel Kraikivski

**Affiliations:** 1 Bioinformatics and Systems Biology Program, School of Bioresources and Technology, King Mongkut’s University of Technology Thonburi, Bangkok, 10150 Thailand; 2 Theoretical and Computational Physics Group, Center of Excellence in Theoretical and Computational Science, King Mongkut’s University of Technology Thonburi, Bangkok, 10150 Thailand; 3 Department of Biological Sciences, Virginia Polytechnic Institute and State University, Blacksburg, VA 24061 USA; 4 Division of Systems Biology, Academy of Integrated Science, Virginia Polytechnic Institute and State University, Blacksburg, VA 24061 USA; 5 VT-Center for the Mathematics of Biosystems, Virginia Polytechnic Institute and State University, Blacksburg, VA 24061 USA

**Keywords:** Stochastic modelling, Cell biology

## Abstract

The cell cycle of budding yeast is governed by an intricate protein regulatory network whose dysregulation can lead to lethal mistakes or aberrant cell division cycles. In this work, we model this network in a Boolean framework for stochastic simulations. Our model is sufficiently detailed to account for the phenotypes of 40 mutant yeast strains (83% of the experimentally characterized strains that we simulated) and also to simulate an endoreplicating strain (multiple rounds of DNA synthesis without mitosis) and a strain that exhibits ‘Cdc14 endocycles’ (periodic transitions between metaphase and anaphase). Because our model successfully replicates the observed properties of both wild-type yeast cells and many mutant strains, it provides a reasonable, validated starting point for more comprehensive stochastic-Boolean models of cell cycle controls. Such models may provide a better understanding of cell cycle anomalies in budding yeast and ultimately in mammalian cells.

## Introduction

Orderly progression through the eukaryotic cell cycle is governed by molecular circuits that control the timely switching from G_1_ into S-G_2_-M and back to G_1_. These transitions typically follow one another in an alternating sequence, but certain disruptions of the control circuits can result in aberrant cell cycles. For example, G_1_-S-G_1_-S and M-(G_1_)-M-(G_1_) cycles are observed in some budding yeast mutant strains^1–3^. Moreover, aberrant cell divisions are common occurrences in cancer cells^4,5^.

Ordinary differential equations (ODEs) are often used to model the molecular control circuits governing cell cycle progression and to explain the irreversible transitions from one cell cycle phase to the next. ODEs have been successfully applied to the complex cell cycle regulatory network in budding yeast^6–8^, as well as specific cell cycle transitions controlled by different checkpoints, e.g., the G_1_/S transition^9^, mitotic exit^10,11^ and the spindle positioning checkpoint (SPOC)^12^. Although ODE-based approaches can provide comprehensive quantitative details, they require accurate estimation of many kinetic parameters in the equations and substantial computational time to simulate large molecular regulatory networks^13^. Furthermore, accounting for stochastic effects within this framework requires additional quantitative data about cell constituents and significantly greater computational resources^14,15^.

To address these difficulties with ODE modeling, many authors have turned to Boolean methods^16–19^. Recently we have adopted a Boolean Kinetic Monte Carlo (BKMC) approach^20^ to explore stochastic Boolean modeling of the budding yeast cell cycle^21^. Although simple (only seven regulatory proteins), the model successfully explained some basic observations of stochastic cell growth and division in wild-type yeast strains; but it was too simple to account for the phenotypes of any mutant strains. Our goal here is to develop a more comprehensive model that addresses in quantitative detail the phenotypes of certain well-characterized mutant strains, including aberrant cycles such as endoreplication and Cdc14 endocycles^22^. The model’s promising results, on a limited subset of experimental data, suggest that our approach to stochastic Boolean modeling may be worth pursuing in more detail in the future.

**Fig. 1:**
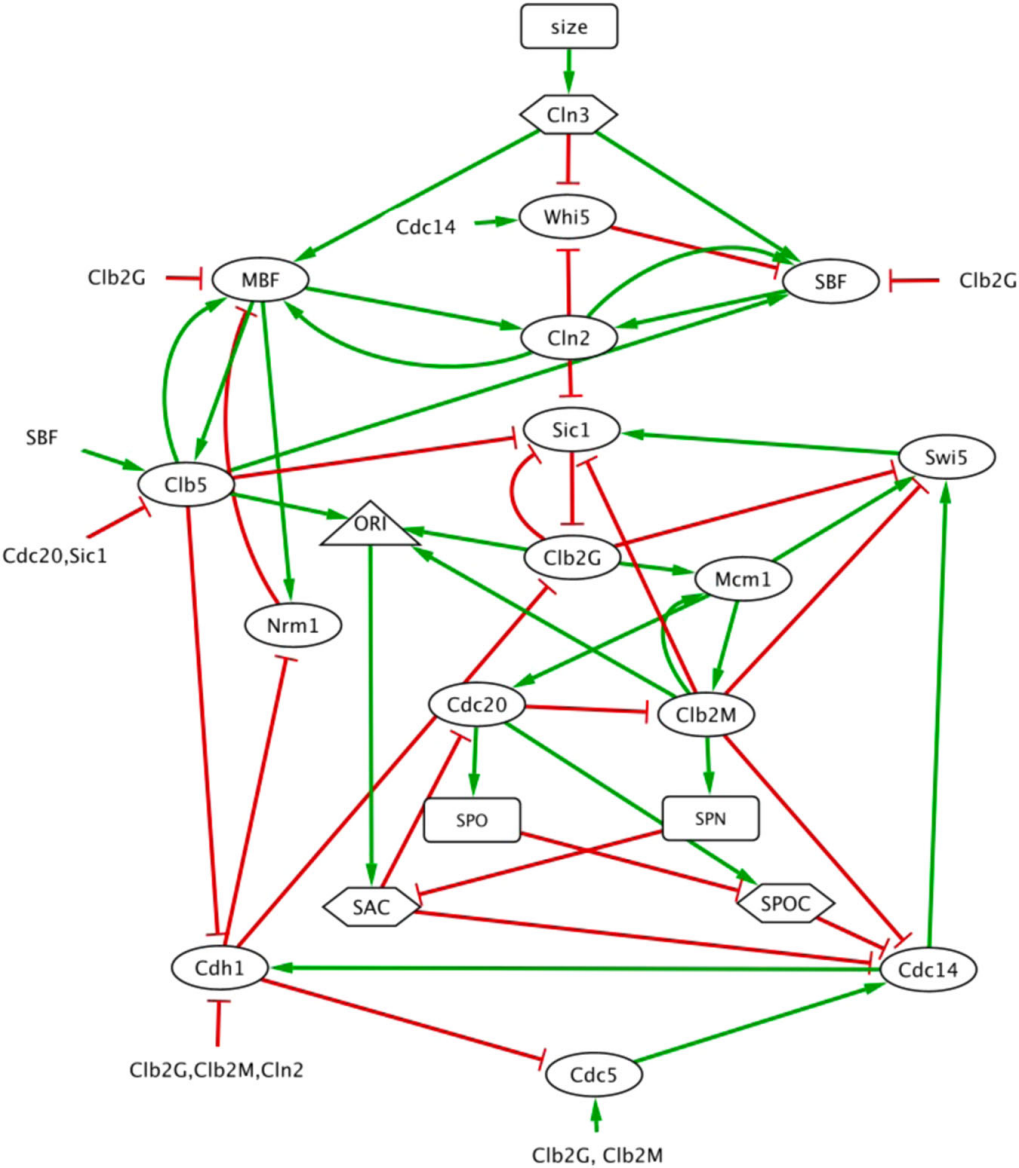
Influence diagram describing our model of cell cycle regulation in budding yeast. The regulatory network consists of nodes connected by edges representing inhibition (red lines with a blunt end) or activation (green lines with a barbed end). Ovals: proteins; hexagons: checkpoints; rectangles: progress variables; triangle: flag variable.

The molecular mechanism of our model (Fig. 1) involves 22 cell cycle-related components: fifteen proteins, three checkpoints, three ‘progress’ variables, and one ‘flag’, as defined in Supplementary Table 1.

## Methods

### Definition of the model

Our model is based on previous work^20,21^ where the Boolean functions and time steps are updated asynchronously and continuously using Gillespie’s stochastic simulation algorithm. We have extended and modified our earlier model in several ways, in order to account for the phenotypes of mutant strains as well as the physiology of wild-type cells. As before, each protein in the model is characterized by a Boolean variable, *X*_*j*,*t*_, where *j* = 1, …, 15 indexes the proteins and *t* ≥ 0 is time. Unlike the BKMC of Stoll et al.^20^, where the Boolean variables are updated according to certain specified ‘reaction propensities,’ we update them by a uniform asynchronous scheme in two steps.

First, we determine which variables might possibly change in the next time step by the following Boolean functions, where the ‘hat’ indicates the ‘potential’ value of *X*_*i*_ at time *t* + Δ*t*:

1 $${\hat{X}}_{i,t+\Delta t}={\rm{Heav}}\left({W}_{i,t}\right)=\left\{\begin{array}{c}1\,{\rm{if}}\,{W}_{i,t} > 0\\ 0\,{\rm{otherwise}}\end{array}\right.,{W}_{i,t}={\omega }_{i0}+\sum _{j}{\omega }_{{ij}}{X}_{j,t},i=1\ldots 15$$

Equation 1 takes as input the 15 Boolean variables representing the proteins, and the 3 Boolean variables representing the states of the checkpoints, in order to calculate an intermediate function, *W*_*i*_(*X*_1_, …, *X*_15_, *Cln3*, *SAC*, *SPOC*), and outputs a Boolean variable, ${\hat{X}}_{i,t+\Delta t}$, the potential update of *X*_*i*_. The ${\omega }_{{ij}}$ coefficients define the Boolean function for updating *X*_*i*_. For example, the Boolean function *X*_1_ = *X*_2_ and *X*_3_ can be implemented by *W*_1_ = − 1.5 + *X*_2_ + *X*_3_, and *X*_1_ = *X*_2_ or *X*_3_ by *W*_1_ = − 0.5 + *X*_2_ + *X*_3_. Similarly, *X*_1_ = *X*_2_ and (not*X*_3_) is equivalent to *W*_1_ = − 0.5 + *X*_2_ – *X*_3_; and *X*_1_ = *X*_2_ or (not *X*_3_) to *W*_1_ = +0.5 + *X*_2_ – *X*_3_. In general, ${\omega }_{{ij}}\, >\, 0$ if variable *j* activates variable *i* (a green barbed arrow in Fig. 1), and ${\omega }_{{ij}} \,<\, 0$ if variable *j* inhibits variable *i* (a red blunt connector in Fig. 1). The ${\omega }_{{ij}}$’s are pure numbers, not rate constants. Their values are chosen once-and-for-all to fix the logical relations in the Boolean dynamics implied by Fig. 1. They are not adjusted to fit quantitative experimental observations (e.g., cycle times, cell size distributions), but they have been adjusted to account for qualitative phenotypes (viable or arrested in a particular phase of the cell cycle) of a panel of mutant strains of budding yeast. In contrast to Boolean modeling with logical functions, we prefer this approach, which is an extension of the method introduced by Li et al.^16^, because the functional form of the *W*_*i*_’s (defined in Eq. 1) is a direct reflection of the network topology in Fig. 1, and the arithmetic calculation is a very compact way to encode a Boolean function. For example, for a node with 4 inputs, there are 2^(2^4) ≈ 65,000 potential Boolean functions, and the one we choose is specified by the relative values of just five coefficients *ω*_*i*0_, *ω*_*i*1_, …, *ω*_*i*4_. However, this approach is limited in that any linear function ${W}_{i,t}={\omega }_{i0}+\sum _{j}{\omega }_{{ij}}{X}_{j,t}$ can be translated into a corresponding logical function for ${\hat{X}}_{i,t+\Delta t}$, but not all possible logical functions can be represented by a linear function. An obvious example is the ‘xor’ function: *X*_1_ = *X*_2_ xor *X*_3_ = (*X*_2_ or *X*_3_) and (not (*X*_2_ and *X*_3_)).

The *W*_*i*_ functions that define our Boolean model for the 15 cell-cycle regulatory proteins are displayed in Table 1. The values of the 65 ${{\omega }_{i0}\text{ and }\omega }_{{ij}}$ coefficients in these functions (for wild-type cells) are specified in Supplementary Table 2A. In Supplementary Table 3 we translate the *W*_*i*_ functions of our Boolean model into traditional logical functions in terms of the elementary relations: and, or, not.

**Table 1:** The *W*_*i*_ functions defining the Boolean network for updating the 15 proteins of the cell-cycle control system

1	${W}_{{\rm{Whi}}5}={\omega }_{{\rm{Whi}}5}+{\omega }_{{\rm{Whi}}5,{\rm{Cdc}}14}\,\cdot\, {Cdc}14-{\omega }_{{\rm{Whi}}5,{\rm{Cln}}2}\,\cdot\, Cln2-{\omega }_{{\rm{Whi}}5,{\rm{Cln}}3}\,\cdot\, Cln3$
2	${W}_{{\rm{SBF}}}={\omega }_{{\rm{SBF}}}-{\omega }_{{\rm{SBF}},{\rm{Whi}}5}\,\cdot\, {Whi}5-{\omega }_{{\rm{SBF}},{\rm{Clb}}2{\rm{G}}}\,\cdot\, {Clb}2G+{\omega }_{{\rm{SBF}},{\rm{Clb}}5}\,\cdot\, {Clb}5+{\omega }_{{\rm{SBF}},{\rm{C}}\mathrm{ln}2}\,\cdot\, {Cln}2+{\omega }_{{\rm{SBF}},{\rm{C}}\mathrm{ln}3}\,\cdot\, {Cln}3$
3	${W}_{{\rm{MBF}}}={\omega }_{{\rm{MBF}}}-{\omega }_{{\rm{MBF}},{\rm{Nrm}}1}\,\cdot\, {Nrm}1-{\omega }_{{\rm{MBF}},{\rm{Clb}}2{\rm{G}}}\,\cdot\, {Clb}2G+{\omega }_{{\rm{MBF}},{\rm{Clb}}5}\,\cdot\, {Clb}5+{\omega }_{{\rm{MBF}},{\rm{C}}\mathrm{ln}2}\,\cdot\, {Cln}2+{\omega }_{{\rm{MBF}},{\rm{C}}\mathrm{ln}3}\,\cdot\, {Cln}3$
4	${W}_{{\rm{Nrm}}1}={\omega }_{{\rm{Nrm}}1}-{\omega }_{{\rm{Nrm}}1,{\rm{Cdh}}1}\,\cdot\, {Cdh}1+{\omega }_{{\rm{Nrm}}1,{\rm{MBF}}}\,\cdot\, {MBF}$
5	${W}_{{\rm{Clb}}5}={\omega }_{{\rm{Clb}}5}-{\omega }_{{\rm{Clb}}5,{\rm{Sic}}1}\,\cdot\, {Sic}1-{\omega }_{{\rm{Clb}}5,{\rm{Cdc}}20}\,\cdot\, {Cdc}20+{\omega }_{{\rm{Clb}}5,{\rm{MBF}}}\,\cdot\, {MBF}+{\omega }_{{\rm{Clb}}5,{\rm{SBF}}}\,\cdot\, {SBF}$
6	${W}_{{\rm{C}}\mathrm{ln}2}={\omega }_{{\rm{C}}\mathrm{ln}2}+{\omega }_{{\rm{C}}\mathrm{ln}2,{\rm{SBF}}}\,\cdot\, {SBF}+{\omega }_{{\rm{C}}\mathrm{ln}2,{\rm{MBF}}}\,\cdot\, {MBF}$
7	${W}_{{\rm{Sic}}1}={\omega }_{{\rm{Sic}}1}+{\omega }_{{\rm{Sic}}1,{\rm{Swi}}5}\,\cdot\, {Swi}5-{\omega }_{{\rm{Sic}}1,{\rm{Clb}}2{\rm{G}}}\,\cdot\, {Clb}2G-{\omega }_{{\rm{Sic}}1,{\rm{Clb}}2{\rm{M}}}\,\cdot\, {Clb}2M-{\omega }_{{\rm{Sic}}1,{\rm{Clb}}5}\,\cdot\, {Clb}5-{\omega }_{{\rm{Sic}}1,{\rm{C}}\mathrm{ln}2}\,\cdot\, {Cln}2$
8	${W}_{{\rm{Cdh}}1}={\omega }_{{\rm{Cdh}}1}+{\omega }_{{\rm{Cdh}}1,{\rm{Cdc}}14}\,\cdot\, {Cdc}14-{\omega }_{{\rm{Cdh}}1,{\rm{Clb}}2{\rm{G}}}\,\cdot\, {Clb}2G-{\omega }_{{\rm{Cdh}}1,{\rm{Clb}}2{\rm{M}}}\,\cdot\, {Clb}2M-{\omega }_{{\rm{Cdh}}1,{\rm{Clb}}5}\,\cdot\, {Clb}5-{\omega }_{{\rm{Cdh}}1,{\rm{C}}\mathrm{ln}2}\,\cdot\, {Cln}2$
9	${W}_{{\rm{Clb}}2{\rm{G}}}={\omega }_{{\rm{Clb}}2{\rm{G}}}-{\omega }_{{\rm{Clb}}2{\rm{G}},{\rm{Cdh}}1}\,\cdot\, {Cdh}1-{\omega }_{{\rm{Clb}}2{\rm{G}},{\rm{Sic}}1}\,\cdot\, {Sic}1$
10	${W}_{{\rm{Clb}}2{\rm{M}}}={\omega }_{{\rm{Clb}}2{\rm{M}}}+{\omega }_{{\rm{Clb}}2{\rm{M}},{\rm{Mcm}}1}\,\cdot\, {Mcm}1-{\omega }_{{\rm{Clb}}2{\rm{M}},{\rm{Cdc}}20}\,\cdot\, {Cdc}20$
11	${W}_{{\rm{Mcm}}1}={\omega }_{{\rm{Mcm}}1}+{\omega }_{{\rm{Mcm}}1,{\rm{Clb}}2{\rm{G}}}\,\cdot\, {Clb}2G+{\omega }_{{\rm{Mcm}}1,{\rm{Clb}}2{\rm{M}}}\,\cdot\, {Clb}2M$
12	${W}_{{\rm{Cdc}}5}={\omega }_{{\rm{Cdc}}5}+{\omega }_{{\rm{Cdc}}5,{\rm{Clb}}2{\rm{G}}}\,\cdot\, {Clb}2G+{\omega }_{{\rm{Cdc}}5,{\rm{Clb}}2{\rm{M}}}\,\cdot\, {Clb}2M-{\omega }_{{\rm{Cdc}}5,{\rm{Cdh}}1}\,\cdot\, {Cdh}1$
13	${W}_{{\rm{Cdc}}20}={\omega }_{{\rm{Cdc}}20}+{\omega }_{{\rm{Cdc}}20,{\rm{Mcm}}1}\,\cdot\, {Mcm}1-{\omega }_{{\rm{Cdc}}20,{\rm{SAC}}}\,\cdot\, {SAC}$
14	${W}_{{\rm{Cdc}}14}={\omega }_{{\rm{Cdc}}14}+{\omega }_{{\rm{Cdc}}14,{\rm{Cdc}}5}\,\cdot\, {Cdc}5-{\omega }_{{\rm{Cdc}}14,{\rm{Clb}}2{\rm{M}}}\,\cdot\, {Clb}2M-{\omega }_{{\rm{Cdc}}14,{\rm{SAC}}}\,\cdot\, {SAC}-{\omega }_{{\rm{Cdc}}14,{\rm{SPOC}}}\,\cdot\, {SPOC}$
15	${W}_{{\rm{Swi}}5}={\omega }_{{\rm{Swi}}5}+{\omega }_{{\rm{Swi}}5,{\rm{Cdc}}14}\,\cdot\, {Cdc}14+{\omega }_{{\rm{Swi}}5,{\rm{Mcm}}1}\,\cdot\, {Mcm}1-{\omega }_{{Swi}5,{\rm{Clb}}2{\rm{M}}}\,\cdot\, {Clb}2M-{\omega }_{{Swi}5,{\rm{Clb}}2{\rm{G}}}\,\cdot\, {Clb}2G$

The second step in updating our model is to identify the change to be made and the time step Δ*t* to be taken. If more than one protein potentially changes state (${\hat{X}}_{i,t+\Delta t}\ne {X}_{i,t}$), then we choose the one that will actually change at random with equal probabilities, i.e., the Boolean model progresses from one state to the next by uniform asynchronous updating. In the BKMC approach, time is updated by choosing Δ*t* from an exponential distribution, parameterized by the total ‘propensity’ (probability per unit time) for any one of the potential changes to occur. This scheme is based on the assumption that each potential change is an elementary chemical reaction^23^, which certainly doesn’t hold in the case of Boolean modeling. If each change (protein synthesis, degradation, phosphorylation, dephosphorylation, etc.) is a series of *k* elementary steps that are independent and identically distributed random variables following an exponential distribution with time constant *θ*, then the total time, Δ*t*, for completion of the process is given by a gamma distribution, with density function:

2 $${\rm{gamma}}\left(\Delta t\right)=\frac{1}{\varGamma \left(k\right){\theta }^{k}}{\left(\Delta t\right)}^{k-1}{e}^{-\frac{\Delta t}{\theta }}$$

The parameters *k* and *θ* determine the mean value of Δ*t* between updates (mean = *kθ*) and its coefficient of variation (CV = $1/\sqrt{k}$). For wild-type cells, we set *k* = 3 and *θ* = 0.3 min (i.e., mean = 0.9 min and CV = 0.58).

In some circumstances, the Boolean model of the protein interactions settles on a steady state (${\hat{X}}_{i,t+\Delta t}={X}_{i,t}$ for all *i* = 1, …, 15), in which case we allow *t* to increase by drawing Δ*t* from a gamma distribution with the same parameter values: *k* = 3 and *θ* = 0.3 min. During the ensuing period, the ‘progress’ variables may change and induce the protein network to leave the steady state and re-enter the cell cycle. For instance, wild-type cells have a G_1_ steady state (*Whi5* = *Cdh1* = *Sic1* = 1, all other Boolean variables = 0). As formalized below, for a cell in this G_1_ steady state, the progress variable *size*_*t*_ steadily increases as the cell grows. When *size*_*t*_ > *S*_0_, the ‘cell size checkpoint’ is satisfied, and the checkpoint variable *Cln3*_*t*_ is changed from 0 to 1. This change kicks the protein interaction network out of the G_1_ steady state and sets the cell division program in motion. In the simulation of some mutants, the protein interaction network falls into a steady state that it cannot leave (i.e., the cell is arrested at some point in the cell cycle), and we stop the simulation after the arrested state becomes evident.

Finally, after the updated protein (say, *X*_*k*_) is chosen and Δ*t* is determined, all the protein variables are updated as follows:

3 $${X}_{k,t+\Delta t}={\hat{X}}_{k,t+\Delta t},{\rm{and}}\,{X}_{i\ne k,t+\Delta t}={X}_{i,t}$$

This completes our description of how the protein variables are updated. The changes we have made to the BKMC scheme of Stoll et al.^20^ precludes using their convenient simulation environment MaBoSS (Markovian Boolean Stochastic Simulator, https://maboss.curie.fr). Instead, we have implemented our scheme in Python and provided all codes on GitHub.

In addition to the Boolean variables tracking the protein interaction network, the model has a Boolean ‘flag’ called ORI and three Boolean ‘checkpoints’ called Cln3, SAC and SPOC. They are updated as explained in the following paragraphs.

- **ORI** specifies the state of the origins of replication on the chromosomes. *ORI* = 0 means the chromosomes are unreplicated and the origins are ‘licensed’ to initiate replication. *ORI* = 1 means that chromosome replication has been initiated and that the origins are now ‘unlicensed’ (i.e., unable to initiate a new round of DNA replication). The value of *ORI* at any time *t* is determined simply by the presence of Clb-dependent kinase activity:
  4 $${{ORI}}_{t}={{Clb}5}_{t}\,{\small{OR}}\,{{Clb}2G}_{t}\,{\small{OR}}\,{{Clb}2M}_{t}$$
- **Cln3** is an indicator of cell growth. *Cln3* = 0 indicates that a cell is too small to start S phase; *Cln3* = 1 means that it has grown large enough to warrant a new round of DNA replication and cell division:
  5 $${C\mathrm{ln}3}_{t}=\text{Heav}({{size}}_{t}-{S}_{0})$$
  ‘Size’ is the progress variable that controls the size checkpoint: *size*_*t*_ > *S*_0_ > 0, where *S*_0_ is the minimum size necessary start the S-G_2_-M sequence. *S*_0_ is a positive random variable assigned to a cell at birth from a lognormal distribution,
  6 $${\rm{lognormal}}\left({S}_{0}\right)=\frac{1}{{S}_{0}\sigma \sqrt{2\pi }}\exp \left(-\frac{{\left(\mathrm{ln}{S}_{0}-\mu \right)}^{2}}{2{\sigma }^{2}}\right)$$
  For a lognormal distribution, ${S}_{\text{mean}}={e}^{\mu }(1+{\frac{1}{2}\sigma }^{2}+\ldots )$ and ${S}_{\text{CV}}=\sigma (1+{\frac{1}{4}\sigma }^{2}+\ldots )$. For simplicity, we encode the lognormal distribution with *μ* = ln(*S*_0_mean_) and *σ* = *S*_0_CV_, which are suitable approximations for our purposes. We chose *S*_0_mean_ = 0.6 so that the size of dividing cells is roughly 1 (arbitrary unit), and *S*_0_CV_ = 0.1 to fit the observed variability (typically ~10%) of size at division. Note: the CV of cell size at bud emergence is ~5%, according to Table S11 of Di Talia et al.^24^.
  During every time step Δ*t*, *size*_*t*_ increases according to:
  7 $${{size}}_{t+\Delta t}={{size}}_{t}{\cdot e}^{r\cdot \Delta t}$$
  where *r* is the specific growth rate of cells. In glucose medium, the mass-doubling time is ~ 90 min, so we choose *r* = 0.0077 min^−1^. At cell division (an event to be defined later), the size of the dividing cell, *size*_@div_, is distributed asymmetrically to the progeny:
  8 $${size}_{{\text{newborn}}\_{\text{mother}}}={f\cdot {size}}_{\text{@div}},{size}_{{\text{newborn}}\_{\text{daughter}}}={\left(1-f\right)\cdot {size}}_{\text{@div}}$$
  We draw *f* from a lognormal distribution with *f*_mean_ = 0.55 and *f*_CV_ = 0.1; these values give a good agreement with the mean and CV of size at birth for both mother and daughter cells growing in glucose medium.
- **SAC**, the ‘spindle assembly checkpoint’, is a Boolean variable indicating the state of alignment of replicated chromosomes on the mitotic spindle:
  9 $${{SAC}}_{t}={\text{Heav}}\left({1-{SPN}}_{t}\right)\,{\small{AND}}\,{{ORI}}_{t}$$
  where *ORI*_*t*_ = 1 indicates that DNA replication has been initiated, and *SPN*_*t*_ tracks the progression of the replicated chromosomes on the spindle; *SPN*_*t*_ = 1 indicating complete alignment. *SPN*_*t*_ is initialized at 0 when the cell enters mitosis, i.e., when Clb2M turns on, and *SPN*_*t*_ increases in each time step thereafter, according to:
  10 $${{SPN}}_{t+\Delta t}={{SPN}}_{t}+\Delta {SPN}$$
  where Δ*SPN* is chosen (in each time step) from a lognormal distribution with parameters *SPN*_mean_ = 0.07 and *SPN*_CV_ = 0.03. Hence, it takes ~14 steps (14∙*kθ* = 13 min) from entry into mitosis until all chromosomes are aligned on the metaphase plate.
- **SPOC**, the ‘spindle position checkpoint’, is a Boolean variable indicating that the fully aligned mitotic spindle is properly positioned in the neck between mother and bud:
  11 $${{SPOC}}_{t}={\text{Heav}}\left({1-{SPO}}_{t}\right)\,{\small{AND}}\,{{Cdc}20}_{t}$$
  where *Cdc20*_*t*_ = 1 indicates that anaphase has been initiated, and *SPO*_*t*_ tracks the movement of the two incipient nuclei during anaphase and telophase. *SPO*_*t*_ = 1 indicates that the bud has received its nucleus. When SPOC turns off, Cdc14 is activated and the cell completes the transition from telophase to G_1_. *SPO*_*t*_ is initialized at 0 when the cell enters anaphase, i.e., when Cdc20 turns on, and *SPO*_*t*_ increases in each time step thereafter, according to:
  12 $${{SPO}}_{t+\Delta t}={{SPO}}_{t}+\Delta {SPO}$$
  where Δ*SPO* is chosen (in each time step) from a lognormal distribution with parameters *SPO*_mean_ = 0.07 and *SPO*_CV_ = 0.03, so it takes ~13 min for the daughter chromosomes to be properly partitioned to the bud.

In addition to the 65 ${{\omega }_{i}\,{\text{and}}\,\omega }_{{ij}}$ coefficients defined in Supplementary Table 2A, the equations defining our model involve 14 adjustable parameters: 4 parameters for updating the SPN and SPO variables, 2 parameters for the specific growth rate (mass doubling time) in glucose and galactose media, 4 parameters to determine the fraction *f* of a dividing cell that is apportioned to the mother cell in glucose and galactose, 2 parameters to determine the critical cell size *S*_0_, and 2 parameters to define Δ*t* for updating the Boolean model. These 14 parameters are manually adjusted to fit the model to experimental observations in both wild-type and mutant strains. See Supplementary Tables 2B-D for their values in wild-type cells.

### Experimental evidence for the Boolean model

Cell cycle progression through G_1_ phase is inhibited by Whi5, which is inactivated (phosphorylated) by Cln3, Bck2, Cln1, and Cln2^25,26^ and activated (dephosphorylated) by Cdc14 phosphatase. In our model (Fig. 1), the ‘*Cln3*’ variable accounts for both Cln3 and Bck2 proteins, and the ‘*Cln2*’ variable represents both Cln1 and Cln2. In G_1_ phase (*Cln3* = *Cln2* = *Cdc14* = 0), Whi5 is active, and it can be inactivated by either *Cln3* = 1 or *Cln2* = 1. Whi5 stays inactive throughout S-G_2_-M and is activated by Cdc14 as the cell exits mitosis, provided either *Cln3* = 0 or *Cln2* = 0. These interactions imply the logical function on Row 1 of Supplementary Table 3:

Whi5 = (notCdc14 and not(Cln2 or Cln3)) or (Cdc14 and not(Cln2 and Cln3)).

This logical function is implemented in our model by the ‘*W*’ function in Row 1 of Table 1.

In late G_1_, Cln3 is activated by cell growth, causing inactivation of Whi5 and subsequent activation of the SBF and MBF transcription factors (Table 1, Rows 2 & 3). Activation of SBF and MBF defines the ‘Start’ transition in the budding yeast cell cycle, after which yeast cells set off on an irreversible path to DNA synthesis, mitosis and cell division. At first, the transcription factors are kept active by Cln3-, Cln2- and Clb5-dependent kinase activities, but later they are inactivated by Clb2-dependent kinase^27–30^. In addition, MBF is regulated by a negative feedback loop with Nrm1 (Table 1, Rows 3 & 4)^31^.

After the Start transition, MBF and SBF activate the synthesis of Clb5, Clb6, and Cln1, Cln2 cyclins, which are responsible for DNA replication (Row 5) and budding (Row 6), respectively^32^. (In our notation, ‘Clb5’ represents both Clb5 and Clb6 cyclins, and ‘Clb2’ represents both Clb1 and Clb2 cyclins.) The origin licensing variable *ORI*, is set to 0 (origins licensed) when both Clb5 and Clb2 are inactivated as a mother cell exits mitosis and divides, then *ORI* is flipped to 1 (DNA replication begins) when either Clb5 or Clb2 is activated in the next cell cycle (Eq. 4).

Once DNA replication is initiated, Clb5 activity promotes the accumulation of active Clb1 and Clb2 cyclins by suppressing Sic1 (Row 7) and Cdh1 (Row 8) in late S phase^33^. Subsequently, a positive feedback loop with Mcm1 transcription factor sets off rapid accumulation of Clb1 and Clb2 cyclins, which drive the cell into M phase^34^ (Rows 9-11). The *Clb2G* variable represents Clb1 and Clb2 cyclin-dependent kinase activities in late S- and G_2_ phases, and *Clb2M* represents their higher activities in M phase. When *Clb2M* = 1, the cell enters M phase and Cdc5 is activated (Row 12).

In M phase, the spindle assembly checkpoint (SAC) prevents the metaphase-to-anaphase transition until all sister chromatids achieve bipolar alignment on the mitotic spindle. The SAC turns ON (*SAC* = 0 → 1) when DNA replication begins (*ORI* = 1) (Eq. 9). Progress in aligning the replicated chromosomes on the mitotic spindle is tracked by the *SPN* variable. When the cell enters M phase (*Clb2M* = 1), the (continuous) variable *SPN*_*t*_ starts to increase (Eq. 10). When *SPN*_*t*_ reaches 1 (i.e., all chromosomes are aligned on the metaphase plate), *SAC* is set to zero.

Once the cell passes the SAC (*SAC* = 1 → 0), Cdc20 is activated (*Cdc20* = 0 → 1) and it promotes the metaphase-anaphase transition. *SPN*_*t*_ is reset to zero, and the Spindle Position Checkpoint (SPOC) is activated (*SPOC* = 0 → 1) to ensure that both mother and daughter cells receive a full set of chromosomes before cytokinesis^35^. Spindle positioning is monitored by the (continuous) *SPO* variable (Eq. 12). When *SPO*_*t*_ = 1, the SPOC is satisfied (*SPOC* = 1 → 0), and *SPO* is reset to 0.

To exit mitosis, Cdc14 must be fully activated (i.e., released from the RENT complex in the nucleolus), which is a consequence of both the FEAR and MEN pathways^36^. FEAR is activated when *SAC* → 0 and MEN when *SPOC* → 0 (Row 14). Finally, Cdc14 activates Cdh1 and Swi5 (Rows 8 & 15), and Swi5 (a transcription factor) activates Sic1 (Row 7). Together Sic1 and Cdh1 reverse the activities of all cyclins. When Clb5, Clb2M and Clb2G activities are destroyed by the combined actions of Cdc20, Cdh1 and Sic1, the mother cell divides according to the rule in Eq. 8, and the newborn cells re-enter G_1_^37^.

### Description of mutant simulations

A mutant strain in which ‘gene K’ is deleted is modeled by setting the Boolean variable for ‘protein K’ = 0 for all time *t* > 0. A mutant strain overexpressing gene L from a GAL promoter is modeled by adding a constant *γ*_*L*_ to the Boolean variable for protein L; i.e., if protein L is chosen to be updated at time t, then Eq. 3 is modified to ${X}_{L,t+\Delta t}={\hat{X}}_{L,t+\Delta t}+{\gamma }_{L}$. Note that this modification may change the logical Boolean function specified by any *W*_*i*,*t*_ for which the sum includes *X*_*L*,*t*_ (which now has the value *γ*_*L*_ or 1 + *γ*_*L*_). Also, for all mutant strains with the GAL promoter, we set *r* = 0.0046 min^−1^ and *f*_mean_ = 0.58.

For mutant strains that exhibit endoreplication and Cdc14 endocycles, we also change the parameters of the gamma distribution (Eq. 2) in order to match the period of oscillation to experimental observations. For example, for the *GAL-CLB2-db*Δ mutant strain, Clb2 (which inhibits Cdh1) is non-degradable and its level is high; therefore, the activation of Cdh1 is delayed compared to wild-type cells with normal levels of Clb2. Also, because Clb2 activates Cdc5, the inactivation of Cdc5 is delayed when the level of Clb2 is high. Therefore, for this mutant strain, the gamma-distribution parameters are set to *k* = 15, *θ* = 1.5 min (mean = 22.5 min and CV = 0.25) for calculating Δ*t* for Cdc5 inactivation and Cdh1 activation. Similar reasoning applies to the *clb1-5*Δ mutant strain that exhibits endoreplication cycles. In the absence of most Clbs, Cdh1 inactivation and Clb6 activation are delayed, and therefore, we set mean = 22.5 min and CV = 0.25 for the timing of these events in this mutant strain.

All parameter changes that are made to model mutant strains are summarized in Supplementary Tables 4 and 5.

## Results

To assess the potential of our method, we present simulations of wild-type cell cycles, of population-level properties of budding yeast cultures, and of mutant strains that exhibit aberrant cycles. We also use the model to predict phenotypes of mutant strains that have not yet been characterized experimentally.

### Simulation of cell cycle progression in wild-type cells

**Fig. 2:**
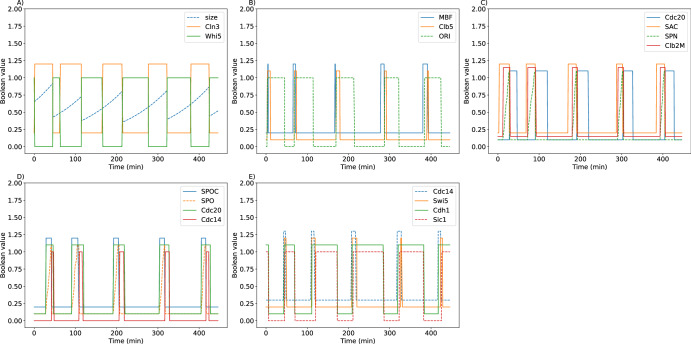
Simulation of wild-type budding yeast cell cycles. Each subplot shows the dynamics of key cell cycle components for five division cycles. **A** Cell size and molecular components that regulate the progression through G_1_. **B** The initiation of DNA replication. **C** Spindle assembly progress in response to the activation of Clb2M. **D** Spindle orientation progress after anaphase. **E** Cdc14 activation and resetting into G_1_. All simulations follow the lineage of mother cells from initial conditions corresponding to stationary G_1_ phase: *Whi5*=*Sic1*=*Cdh1* = 1, all other variables = 0, and size=0.65 (the average size of a mother cell just after division). Some of the variables are offset for clearer visualization: *Clb5*, *SPN*, *Clb2M*, *SPO*, *Cdc20*, and *Cdh1* are offset by 0.1; *Cln3*, *MBF*, *SAC*, *SPOC* and *Swi5* are offset by 0.2; and *Cdc14* is offset by 0.3.

Figure 2 shows simulations of key components regulating cell cycle events in wild-type budding yeast. Newborn yeast cells must grow to a ‘critical size’ in order to activate Cln3 and subsequently to inactivate Whi5, which then permits activation of SBF and MBF transcription factors (Fig. 2A). MBF induces the synthesis of Clb5, which induces DNA replication (*ORI* = 1 identifies the onset of S phase, Fig. 2B). The spindle assembly progress variable (*SPN*) indicates progression through G_2_/M into metaphase. When *SPN* ≥ 1, the spindle assemble checkpoint variable (*SAC*) is set to zero, which allows the activation of Cdc20 and the cell to progress into anaphase (see Fig. 2C).

The spindle-orientation progress variable (*SPO*) accounts for proper segregation of chromosomes into mother and daughter cell compartments during anaphase. When *SPO* ≥ 1, the spindle orientation checkpoint (*SPOC*) is set to zero, which allows Cdc14 to be fully released from the nucleolus (Fig. 2D). Once Cdc14 is released, it activates Cdh1 and Swi5 (which initiates synthesis of Sic1), thereby resetting the cell back to G_1_ (Fig. 2E).

Model simulations of wild-type cells are very robust with respect to perturbations of the 65 *ω*_*ij*_ coefficients in Supplementary Table 2A. Each one of them can be perturbed by at least 40% and the model predicts ‘viable’ wild-type cell cycles (see Supplementary Fig. 2A). When three specific coefficients (*ω*_Mcm1,Clb2G_, *ω*_Clb2M,Mcm1_ and *ω*_Cdc20,Mcm1_,) are reduced by 50% (Supplementary Fig. 2B), the model fails to yield successful division cycles because, in each case, a crucial component (Mcm1, Clb2M and Cdc20, respectively) fails to activate.

**Fig. 3:**
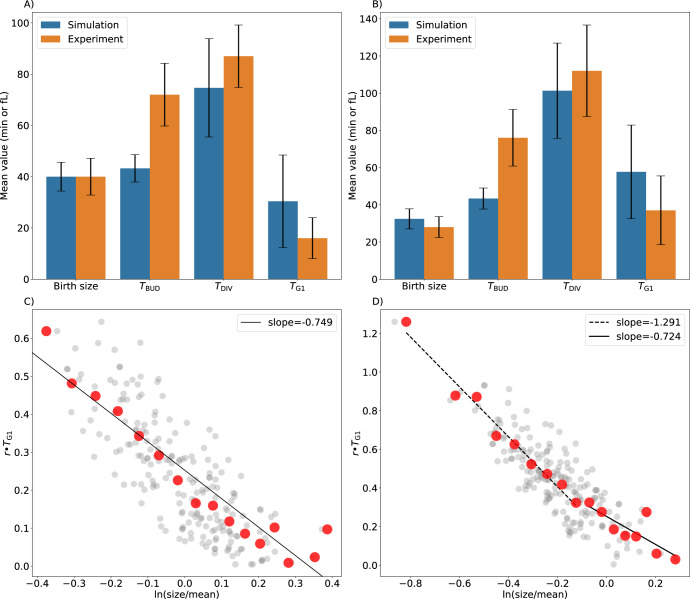
Population-level statistics from model simulations and experimental observations. The mean values and coefficients of variation for four cell-cycle properties in populations of mother cells (**A**) and daughter cells (**B**). To transform dimensionless cell size into volume in fL, we used a conversion factor of 75 fL, which was derived by equating the mean size of mother cells to the experimental mean volume of ~40 fL. To visualize the joint distributions of size at birth and G_1_ duration (*T*_G1_) in mother cells (**C**) and daughter cells (**D**), we plot (grey dots) 200 simulated cells sampled from the whole population. To estimate the trends in the data, we plot (red dots) the average value of the grey dots in bins of size 2 fL, exactly as implemented by the authors of the experimental data^24^.

Next, the model is used to simulate the exponential expansion of a population of budding yeast cells, in order to estimate the means and standard deviations of observable cell-cycle measures: the period from birth to division (*T*_c_), the duration of G_1_ phase from mitotic exit to S phase (*T*_G1_), the period from budding to division (*T*_bud_), and cell size at birth. We compare these simulation results with corresponding experimental data from ref.^24^. in Fig. 3. Overall, the model accurately simulates these population-level properties in both mother cells (Fig. 3A) and daughter cells (Fig. 3B), although the model overestimates *T*_G1_ variability in both mother and daughter cells. Similar discrepancies were observed in simulations based on previous models^21,38^. Figure 3C and D show the joint distributions of size-at-birth and *T*_G1_ for mother and daughter cells. As in ref.^24^, we plot *r* ∙ *T*_G1_ vs. ln(size/mean), where (in glucose medium) *r* = 0.0077 min^−1^ and ‘mean’ = mean size of mother cells at birth = 40 fL = 0.55 in units of dimensionless size. The simulated data points are fitted with trendlines, as was done to analyze the experimental data^24^. Although the estimated slopes of the trendlines for mother cells ( − 0.74) and daughter cells (large −0.72 & small −1.29) show similar trends to the experimental data (slope = −0.1 for mother cells; −0.3 & −0.7 for large & small daughter cells, respectively), the theoretical slopes are considerably more negative than observations warrant, suggesting that size-control in the model is considerably stronger than in reality.

**Fig. 4:**
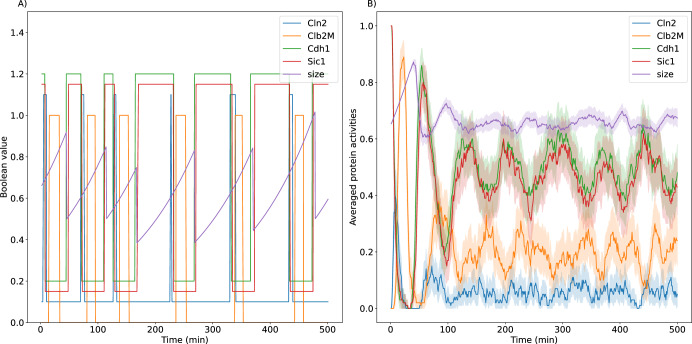
Loss of synchrony of protein activities from model simulations. **A** A single cell is tracked over time from one division to the next. *Cdh1*, *Sic1* and *Cln2* are offset by 0.2, 0.15 and 0.1, respectively. **B** Many such simulations are combined to predict the loss of synchrony in a population of cells. Solid line = average activity, vertical bar indicates ± 95% Confidence Interval.

To estimate how fast a simulated population of cells loses synchrony over time, 100 cells with average size of 0.65 were initiated in the G_1_ state and tracked (both mother and daughter cells) for 500 min (Fig. 4A). Cell size and protein activities of all cells extant at time *t* were averaged and the results plotted as functions of *t* (Fig. 4B). The population-average results clearly show a loss of synchrony that agrees well with observations^39^. Protein activities and cell size quickly lose synchrony due to the unequal division of material between daughter and mother cells.

Some of the mutant strains discussed in later sections are grown in galactose medium, rather than glucose. To simulate this change of growth medium, we change the specific growth rate *r* to 0.0046 min^−1^ (mass doubling time = 150 min, typical for growth on galactose) and the division fraction *f*_mean_ to 0.58 (cell division is more asymmetric in poorer growth medium). With these changes, the interdivision times in galactose medium for mother and daughter cells are computed to be 113 min and 184 min, respectively, in good agreement with the observations of Lord and Wheals (1980).

### Simulation of known mutant phenotypes

To test the accuracy of our model in accounting for mutant phenotypes, we simulated cell cycle progression in 48 experimentally characterized mutant strains, including gene deletion and overexpression mutants. Of these strains, our model agrees with 40 observed phenotypes; see Supplementary Table 4. The model’s success rate for predicting phenotypes of mutant strains depends on our choice of the 65 *ω*_*ij*_ parameters in Supplementary Table 2A, because these parameters determine the precise logical functions at play in wild type and mutant cells. We have obtained a success rate of 83% by manual adjustment of the parameters. Presumably a higher success rate could be achieved by an automated parameter estimation procedure, but we defer this step to later developments of the modeling approach.

**Fig. 5:**
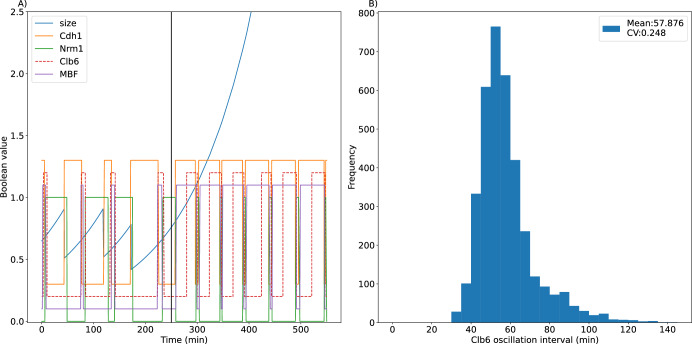
Simulation of endoreplication cycles in the *clb1-5*Δ mutant strain. **A** Wildtype cell cycles for *t* < 250 min, and endoreplication cycles for *t* > 250 min. *Cdh1*, *Clb6* and *MBF* are offset by 0.30, 0.20, and 0.10, respectively. **B** The distribution Clb6 oscillation periods.

In this subsection, we focus on mutant strains exhibiting aberrant cycles. For the *clb1-5*Δ strain, in which all Clbs—except Clb6—are deleted, cells replicate the genome multiple times without mitosis^3^, a phenotype called endoreplication. Because the Clb5 variable represents both Clb5 and Clb6, the action of Clb6 in *clb1-5*Δ mutant is simulated by reducing the basal parameter *ω*_Clb5_ to −1.1, and the values of parameters *ω*_*i*,Clb5_ (which describe the influence of Clb5 and Clb6 on target protein *i*) were reduced by eight-fold (see Supplementary Table 4). Because MBF-induced transcription of the *CLB6* gene is slower than the *CLB5* gene, the accumulation of Clb6 protein is delayed by 22.5 min compared to 0.9 min in wild type cells. Also, due to absence of most Clbs in the *clb1-5*Δ mutant, Cdh1 inactivation is delayed by 22.5 minutes compared to 0.9 minutes in wild type. As Fig. 5A shows, after *t* = 250 min when all Clbs except Clb6 are deleted, the *clb1-5*Δ mutant fails to enter mitosis and to divide, and cell size becomes extremely large. However, MBF, Clb6, Cdh1 and Nrm1 continue to oscillate, driven by the negative feedback loop MBF → Clb6 –| Cdh1 –| Nrm1 –| MBF. Figure 5B shows the distribution of endoreplication periods. The estimated period of Clb6 oscillations, 57.9 ± 14.3 min, is in good agreement with experimental observations^3^.

The mean delays we have introduced (22.5 min for the accumulation of Clb6 and the inactivation of Cdh1) are somewhat arbitrary. The experimental observation (that the period of endoreplication is ~60 min) only fixes the total delay to be ~45 min (see Supplementary Fig. 3), which we split evenly between the two processes.

Endoreplication cycles in our model disappear when any component (i.e., Cdh1, Nrm1, MBF or Clb6) in the negative feedback loop is deleted. In addition, Cln3, Cln2, and SBF are essential for endoreplication cycles. Further, we tested all pairwise deletions of model components in *clb1-5*Δ mutant strain to identify other mutant strains that might exhibit endoreplication cycles. Mutant strains that lose or retain endoreplication cycles are shown in Supplementary Fig. 1A.

At this point we note that, despite the fact that MBF is a transcription factor for Nrm1 (Fig. 1), our logical function for updating Nrm1 (Supplementary Table 3, row 4) is Nrm1 = notCdh1, independent of whether MBF is active or not. In other words, with our choice of *ω*_Nrm1_ = 0.5, even if MBF = 1, Nrm1 cannot accumulate if Cdh1 = 1. If we were to choose *ω*_Nrm1_ = 1.5, then the logical function would be Nrm1 = MBF or notCdh1, because MBF-induced expression of Nrm1 would overwhelm its degradation initiated by Cdh1. In this case (*ω*_Nrm1_ = 1.5), the *clb1-5*Δ mutant strain still exhibits endoreplication, but the distribution of Clb6 oscillation periods is bimodal (Supplementary Fig. 4). In these mutant cells, Cln3 = 1, Clb2G = Clb2M = 0, and *ω*_MBF,Clb5_ = 3/8; hence, *W*_MBF_ > 0 if Nrm1 = 0 and < 0 if Nrm1 = 1, so MBF = notNrm1. In this case, when Cdh1 = 1, the regulatory network has a short, negative feedback loop (Nrm1 = MBF, MBF = notNrm1) that, under asynchronous updating, can oscillate a few times (Nrm1 on, MBF off, Nrm1 off, MBF on, …) before MBF is selected to turn on Clb6 and the cell proceeds to endoreplicate. This effect is evident in the temporal simulations of MBF and Nrm1 in Supplementary Fig. 5. With our preferred choice of *ω*_Nrm1_ = 0.5, the short, negative feedback loop is broken and the endoreplication cycles are more uniform (see Supplementary Fig. 6). Furthermore, with the alternative parameter choice (*ω*_Nrm1_ = 1.5), in the strain *clb1-5*Δ *cln2*Δ, Cdh1 is constitutively active and the short NFL is persistently in play; hence, the cells exhibit rapid endoreplication cycles with a period of 6 updates. To avoid these unlikely results, we prefer the choice *ω*_Nrm1_ = 0.5 and the logical function Nrm1 = notCdh1.

**Fig. 6:**
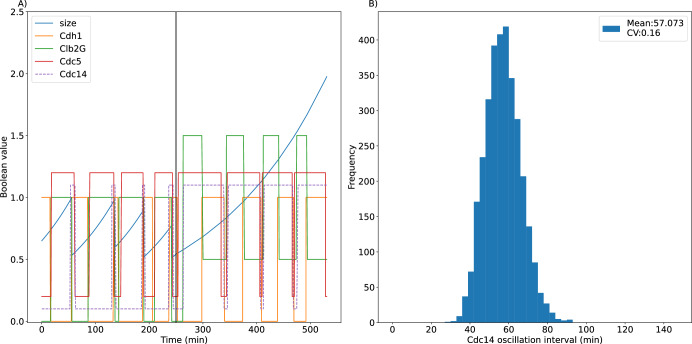
The simulation of Cdc14 endocycles in the *GAL-CLB2-db*Δ mutant strain. **A** Wildtype cell cycles for *t* < 250 min, and Cdc14 endocycles for *t* > 250 min. As before, *Cdc5* and *Cdc14* are offset by 0.20 and 0.10, respectively, for clearer visualization. On the other hand, *Clb2G* (the green curve) is not offset for plotting purposes but rather because Clb2 protein is overproduced in this mutant strain. **B** The distribution of Cdc14 oscillation periods.

Another mutant strain that exhibits aberrant cycles is *GAL-CLB2-db*Δ. The high level of non-degradable Clb2 causes cell cycle arrest in mitosis. Although the cells cannot exit mitosis, the high level of Clb2 supports the activation of Cdc5 which promotes Cdc14 release, Cdc14 then activates Cdh1 which degrades Cdc5. This negative loop (Cdc5 → Cdc14 → Cdh1 –| Cdc5) results in Cdc14 endocycles (Fig. 6A). Because Clb2 activity is high in this mutant strain, the time delays for inactivation of Cdc5 and for activation of Cdh1 are increased to 22.5 min. Figure 6B shows the distribution of endocycle periods. The averaged period of Cdc14 oscillations is 57.1 ± 9.1 min, in agreement with experimental observations^1,2^. Supplementary Figure 1B shows that Cdc14 endocycles depend not only on *CDC5*, *CDH1* and *CDC14* genes, as expected, but also on *CLB2*, *MCM1* and *CDC20* genes, and, furthermore, that the double-deletion strain, *cdc20Δ mcm1Δ*, restores Cdc14 endocycles. The genetic dependencies of Cdc14 endocycles are described as follows.

1. *CDC20*. Clb2M activity oscillates between 0 and 1 during normal cell cycles.
2. *cdc20Δ*. *Clb2M* = 1, and the cell cannot exit mitosis because Cdh1 and Sic1 are inhibited.
3. *GAL-CLB2-dbΔ*. Clb2M activity oscillates between 0.5 and 1.5, which supports Cdc14 endocycles but blocks return to G_1_.
4. *GAL-CLB2-dbΔ cdc20Δ*. Clb2M activity is constitutively high in this strain because Mcm1 is constitutively active, and *Clb2M* = 1.5 blocks Cdc14 endocycles by inhibiting Cdh1 and activating Cdc5.
5. *GAL-CLB2-dbΔ cdc20Δ mcm1Δ*. In the absence of both Mcm1 and Cdc20, *Clb2M* = 0.5, which is a ‘sweet spot’ for Cdc14 endocycles.

### Model predictions

**Fig. 7:**
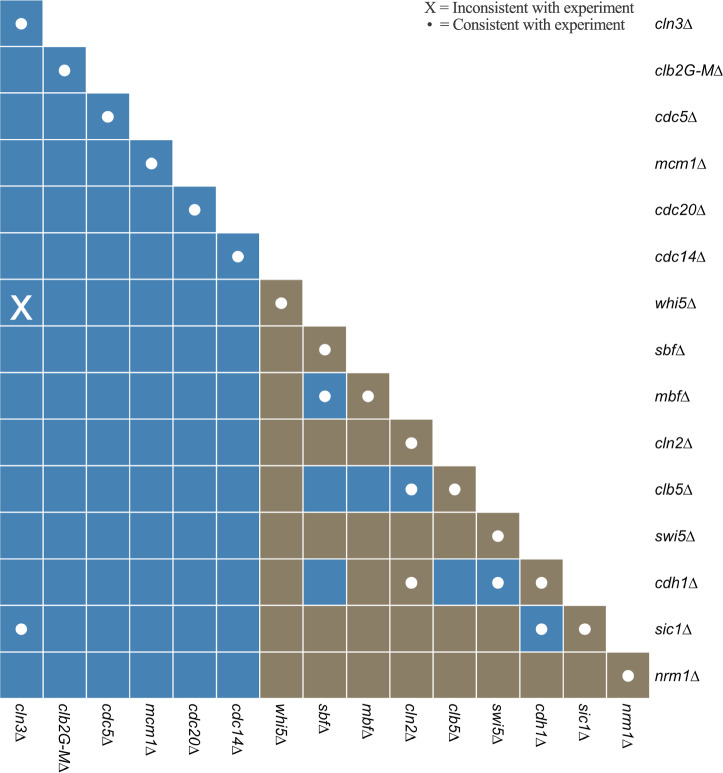
Predicted phenotypes of double-deletion strains. Each rectangular cell corresponds to a combination of two deleted components listed along the vertical and horizontal axes. Blue = inviable, brown = viable phenotype. A white dot indicates that the prediction is consistent with experimental observations, and X indicates a difference between the simulated and observed viability. The elements along the diagonal correspond to single-deletion strains.

Next, we used the model to predict the viability of mutant strains with single and double deletions of model variables. Because some model variables already represent two genes (MBF = SWI6 + MBP1, SBF = SWI6 + SWI4, CLN3 = CLN3 + BCK2, CLN2 = CLN1 + CLN2, CLB5 = CLB5 + CLB6, and CLB2 = CLB1 + CLB2), combining their deletions with the deletion of other model components generates triple- or quadruple-deletion strains of budding yeast. The model, with 15 genetic components, correctly predicts the viability/inviability of the 15 ‘single’ deletion strains (Fig. 7). Of the 15×14/2 = 105 ‘double’ deletion strains, only seven combinations have been characterized experimentally, and the model correctly predicts six of them; only the *cln3*Δ *bck2*Δ *whi5*Δ strain is incorrectly predicted to be inviable (see also, row 26 of Supplementary Table 4). The viability of this strain indicates that the only essential function of Cln3+Bck2 is to inactivate Whi5, but in our model, even when *Whi5* = 0, Cln3 must be activated (by cell growth) in order to activate SBF and MBF at Start. In the cell (but not in our model), this role of Cln3 is most likely backed up by Cln2.

The model predicts that eight double-deletion strains are synthetically lethal (the eight blue squares in the brown ‘background’ in Fig. 7). Four of these strains are already known to be synthetic lethal; the other four combinations, which are genuine predictions of the model, are described below.

1. *cdh1Δ clb5Δ*. In *cdh1Δ*, SBF compensates for the inhibition of MBF by Nrm1. In *clb5Δ*, Cln2 is available to inhibit Cdh1 and Sic1. In *cdh1Δ clb5Δ*, Nrm1 and Sic1 are permanently active, and Cln2 and Clb2 are permanently repressed.
2. *cdh1Δ sbfΔ*. In *cdh1Δ*, SBF compensates for the inhibition of MBF by Nrm1. In *sbfΔ*, MBF drives Clb5 synthesis and cell cycle progression. In *cdh1Δ sbfΔ*, SBF is absent and MBF is inhibited by Nrm1, so the cell arrests in G_1_.
3. *clb5Δ sbfΔ*. In *clb5Δ*, Cln2 is available to inhibit Cdh1 and Sic1. In *sbfΔ*, MBF drives Clb5 synthesis and cell cycle progression. In *clb5Δ sbfΔ*, neither Clb5 nor Cln2 are available to inactivate Cdh1 and Sic1, so the cell is arrested in G_1_.
4. *clb5Δ mbfΔ*. In *clb5Δ*, Cln2 is available to inhibit Cdh1 and Sic1. In *mbfΔ*, SBF drives Clb5 synthesis and cell cycle progression. Because Cln2 synthesis requires both SBF and MBF activities in our model, the *clb5Δ mbfΔ* strain, lacking both Clb5 and Cln2, cannot inactivate Cdh1 and Sic1, so cells arrest in G_1_.

These explanations suggest that the synthetic lethal strains, *cdh1Δ clb5Δ* and *cdh1Δ sbfΔ*, should be rescued by further deletion of *NRM1*. The triple mutants, *cdh1Δ clb5Δ nrm1Δ* and *cdh1Δ sbfΔ nrm1Δ*, are indeed viable in our model simulations (Supplementary Fig. 7).

## Discussion

In this study, we developed a stochastic Boolean model of the budding yeast cell cycle that can correctly explain both normal cell cycle progression and aberrant cycles (endoreplication and Cdc14 endocycles). Understanding the mechanism of aberrant cycles and identifying controls that suppress these cycles in wild-type cells is important because these cycles are found in many types of cancer^40^. Our model suggests that endoreplication cycles are driven by the negative feedback loop MBF → Clb6 –| Cdh1 –| Nrm1 –| MBF, and that Cdc14 endocycles are driven by the negative loop Cdc5 → Cdc14 → Cdh1 –| Cdc5. Although our model has not yet been optimized to account for mutant phenotypes, it already accounts for the viability/inviability of 40 gene deletion and overexpression mutant strains out of 48 strains tested. Furthermore, we used the model to predict the phenotypes of 120 mutant strains carrying one-, two-, three- or four gene deletions.

The stochastic Boolean model proposed here is closely related to the stochastic differential equation (SDE) approach proposed in Laomettachit et al.^38^ and applied in great detail to the budding yeast cell cycle in Kraikivski et al.^8^. At the core of the SDE approach is a nonlinear ODE model of a protein signaling network:

13 $$\begin{array}{l}\frac{1}{{\beta }_{i}}\frac{d{Y}_{i}}{{dt}}=H\left({\sigma }_{i}{W}_{i}\right)-{Y}_{i},{\text{where}}\,{W}_{i}={\omega }_{i0}+\mathop{\sum}\limits_{j}{\omega }_{{ij}}{Y}_{j},{\text{and}}\,H\left(\sigma x\right)=\frac{1}{1\,+\,{e}^{-\sigma x}}\end{array}$$

where *β*_*i*_, *σ*_*i*_, and *ω*_*ij*_ ‘s are parameters. Equations 1 and 13 are closely related because *H*(*σx*) → Heav(*x*) as *σ* → ∞. With this identification in mind, it would be (relatively) easy to translate a stochastic Boolean model (in the format proposed here) to a continuous, nonlinear SDE model of the type proposed by Laomettachit et al.^38^. Indeed, some predictions by Kraikivski et al.^8^ are consistent with our stochastic Boolean model: e.g., the *cln1*∆ *cln2*∆ *swi6*∆ *mbp1*∆ strain is predicted to be viable, whereas the *cln3*∆ *bck2*∆ *cdh1*∆ strain is inviable.

It is instructive to compare our model to the Boolean models of Li et al.^16^, Irons^19^ and Fauré et al.^18^, see Supplementary Table 6. Li et al.^16^ were primarily concerned with demonstrating the ‘robustness’ of cell cycle progression: Start → DNA synthesis & budding → prophase-metaphase-anaphase-telophase → early G_1_ arrest. In addition to emphasizing the robustness of the cell-cycle control system, Irons focused on ‘sub-network analysis,’ identifying a ‘core network’ (his Fig. 6C) that is very similar to the four-variable ODE model of Battogtokh and Tyson^41^ (their Fig. 5, where their ‘Hct1’ plays the same role as Iron’s ‘CKI’). Fauré et al.^18^ were concerned to show how simple logical models could be ‘composed’ into a single ‘comprehensive’ model that rivals a detailed ODE model^6^ in terms of successfully simulating mutant cell phenotypes. We are primarily concerned with modifying the Boolean approach to allow for stochastic modeling in terms of real time and real cell-size variables, in order to compare model simulations with experimental data, as well as to simulate the phenotypes of ~150 mutant strains (40% known experimentally and 60% novel predictions).

All four models follow the time evolution of 10 dynamical variables representing the core cell-cycle regulatory proteins Cdh1, Sic1, Cln3/Bck2, SBF/MBF, Cln1/2, Clb5/6, Mcm1, Swi5, Clb1/2 and Cdc20. Irons adds Cdc14, Yhp1/Yox1 (a transcriptional repressor of Cln3), and two ‘pathways’ FEAR and MEN. Fauré et al.^18^ ignore Yhp1/Yox1 and unpack the FEAR and MEN pathways. We add Whi5, Nrm1 and Cdc5. Like Li et al.^16^, we define the protein interaction network through Heav(*W*_*i*_) functions, whereas Irons and Fauré et al.^18^ use logical functions. Li/Irons/Fauré update their models synchronously and deterministically in discrete time steps, whereas we update our model asynchronously and stochastically in real time (min). Irons introduces ‘dummy’ variables to simulate time delays in protein synthesis and degradation and in cell cycle events; Fauré et al.^18^ achieve similar effects by assuming that ~40% of their proteins (e.g., Clb2, Clb5 and Cdc20) are multi-state variables (2 or 3 ‘active’ states). We have only one multi-state variable: Clb2G (low activity of Clb2 in G_2_ phase) and Clb2M (higher activity form in M phase). We account for time delays by choosing Δ*t* (updating intervals) from a gamma distribution with mean and CV chosen to fit observed temporal progression through the cell cycle in wild-type and endocycling mutants. Only our model has a real variable ‘size(t)’ to track cell growth. To track cell growth and division, Fauré introduced two multistate variables, called ‘MASS’ and ‘CYTOKINESIS,’ which were updated separately from the other variables by a set of ‘priority rules.’ Li’s model goes through a sequence of 13 states from Start (activation of Cln3) to ‘Stationary G_1_’ (*Cdh1* = *Sic1* = 1, all other variables = 0), and it ‘cycles’ only if *Cln3* = 0 in Stationary G_1_ is flipped to 1 ‘externally’ (say, by cell growth). Iron’s model goes through a repetitive sequence of 19 states, driven by a negative feedback loop, Cln3 → SBF/MBF → Yhp1/Yox1 **−|** Cln3, with a lengthy time delay. Fauré’s model has a 22-state cycle because the activation of MASS in early G_1_ phase drives the Start transition. Li et al.^16^ discussed checkpoints briefly but did not consider mutant phenotypes. Irons’ model is consistent with the phenotypes (viable/inviable) of 13 deletion mutants and with 4 types of checkpoints (‘Start,’ ‘morphogenesis,’ ‘spindle assembly,’ and ‘DNA damage’). Fauré et al.^18^ successfully simulated 135 mutant strains by defining alternative logical rules for particular components of the network, to account for the genetic changes in each mutant strain. Using our model, we analyzed both deletion and overproduction mutants (correctly predicting the phenotypes of 40/48 strains) and simulated mutant strains exhibiting endoreplication (multiple rounds of DNA replication) and Cdc14 endocycles (multiple attempts at mitotic exit). We also predicted the phenotypes of ‘double deletion’ mutants: of 105 such strains, 9 phenotypes are known experimentally, and our model agrees with 8 of them. In addition, our model is stochastic, so it can be used to predict cell-cycle time distributions, cell size distributions, and correlations between birth size and time spent in G_1_ phase. Our model simulations are in good agreement with most of these statistical measures.

When updating Boolean models asynchronously, there are subtle timing issues that must be recognized. In our approach (which was motivated by Gillespie’s stochastic simulation algorithm), we first identify those variables that potentially change in the next iteration and then choose one of them at random to actually change. Alternatively, in each iteration one might choose at random any variable to be updated, whether or not it changes state. The latter method avoids certain unrealistic consequences of the former method; e.g., the period of a negative feedback oscillator (in terms of number of iterations) may depend on how other variables could potentially change state, whether or not the ‘other variables’ are causally connected to the oscillator variables. For example, in our model, Cdc5, Cdc14 and Cdh1 are engaged in a negative feedback loop, but potential oscillations of this loop are suppressed by the overpowering cycle of ClbM activation during normal cell division cycles. However, in the *GAL-CLB2-dbΔ* mutant strain, the constitutively high activity of ClbM allows the Cdc5-Cdc14-Cdh1 NFL to oscillate freely, albeit at an unrealistically high frequency. To match the Cdc14 oscillation period to observations, we ‘renormalize’ the transition times by increasing the mean delays of the gamma distributions. This parameter change is, in a sense, the price we pay to fit a simple, stochastic Boolean model to experimental data in real time.

Our modeling approach supplements the simplicity of Boolean models with quantitative details, such as real continuous time, and with easily interpretable, adjustable parameters that are helpful in accounting for mutant phenotypes. Therefore, our approach lies somewhere between Boolean models that lack quantitative details necessary to explain experimental observations and ODE models that provide all these details at the expense of estimating many obscure kinetic rate constants. In our approach, only the parameters that determine the delays must be estimated by fitting model simulations to experimental data. Also, our approach incorporates stochastic effects at minimal computational cost.

## Supplementary information

Supplemental Information

## Data Availability

The generated data for reproducing all figures in this study are available on GitHub: https://github.com/Ktaoma/A-continuous-time-Boolean-model-of-the-endocycle-events-in-budding-yeast.

## Code availability

The model is available in BioModels^42^ with the identifier MODEL2405030001 (https://www.ebi.ac.uk/biomodels/MODEL2405030001). Computer codes to simulate all figures are available on GitHub: https://github.com/Ktaoma/A-continuous-time-Boolean-model-of-the-endocycle-events-in-budding-yeast.
