# Supplementary material for: Stochastic Boolean model of normal and aberrant cell cycles in budding yeast
Source: NPJ Syst Biol Appl. 2024 Oct 18;10:121. doi: 10.1038/s41540-024-00452-3 (PMC11487276; doi:10.1038/s41540-024-00452-3)
Supplement: Supplementary file 1 — Supplemental Information [file 41540_2024_452_MOESM1_ESM.pdf]

Supplementary Information

Stochastic Boolean Model of Normal and Aberrant Cell Cycles in Budding Yeast

by

Kittisak Taoma, John J. Tyson, Teeraphan Laomettachit & Pavel Kraikivski

Supplementary Figure 1. Predicted phenotypes when additional deletions are added to mutant strains that exhibit endocycles.

Supplementary Figure 2. Robustness of wild-type cell viability with respect to perturbations of the 65 ω_{ij} coefficients from the values given in Supplementary Table 2.

Supplementary Figure 3. Endocycle period depends linearly on the mean delays ($\Delta t_{\mathrm{mean}} = k \cdot \theta$) of the two slow steps in the negative feedback loop.

Supplementary Figure 4 & 5. Simulation of endoreplication cycles in the *clb1-5*Δ mutant strain with $\omega_{\mathbf{Nrm1}}$ = 1.5.

Supplementary Figure 6. Simulation of endoreplication cycles in the *clb1-5*Δ mutant strain with $\omega_{\mathbf{Nrm1}}$ = 0.5.

Supplementary Figure 7. Simulation of the triple mutants *cdh1Δ clb5Δ nrm1Δ* and *cdh1Δ sbfΔ nrm1Δ*.

Supplementary Table 1: Model variables associated to molecular components of the budding yeast cell-cycle control system in Figure 1. CDK, cyclin-dependent kinase.

Supplementary Table 2: Model parameter values.

Supplementary Table 3: The logical functions for the Boolean network used to update the 15 proteins of the cell-cycle control system.

Supplementary Table 4: Mutant strains simulated by the model.

Supplementary Table 5: Summary of time-delay parameters in endoreplication cycles and Cdc14 endocycles.

Supplementary Table 6. Comparison of four Boolean models of the budding-yeast cell-cycle engine.

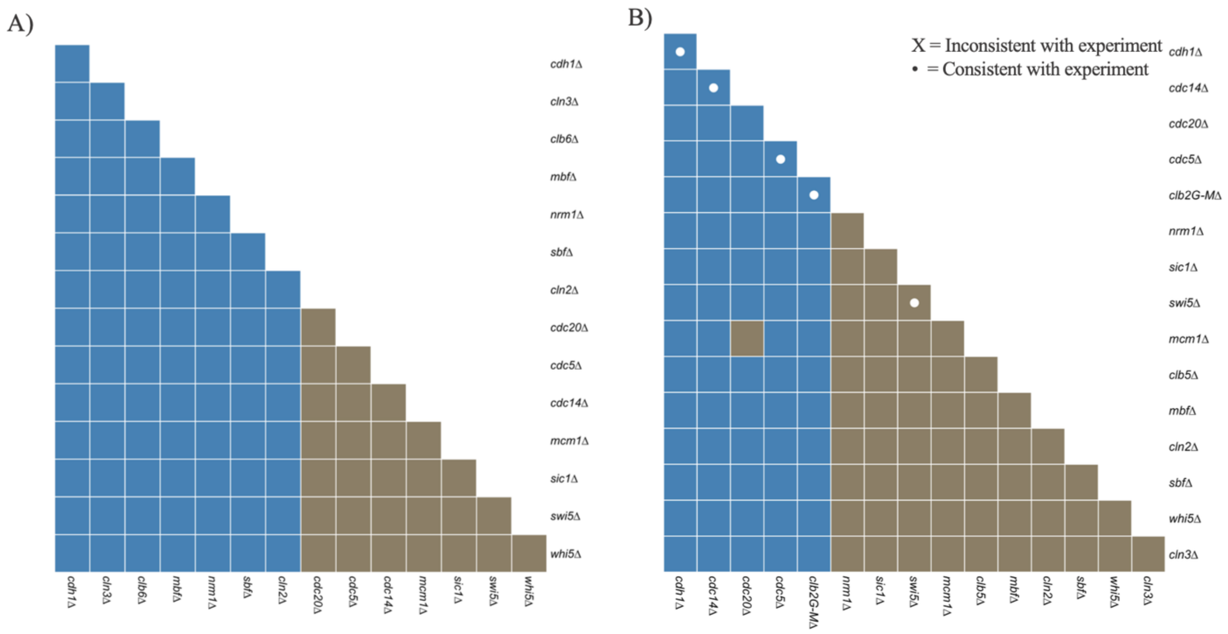

Supplementary Figure 1. Predicted phenotypes when additional deletions are added to mutant strains that exhibit endocycles. (A) The endoreplication strain *clb1-5*Δ. (B) The Cdc14 endocycle strain GAL-*CLB2-db*Δ. (Blue) Mutants that lose endo-oscillations. (Brown) Mutants that retain endo-oscillations. (White dot) Simulations that are consistent with experimental data.

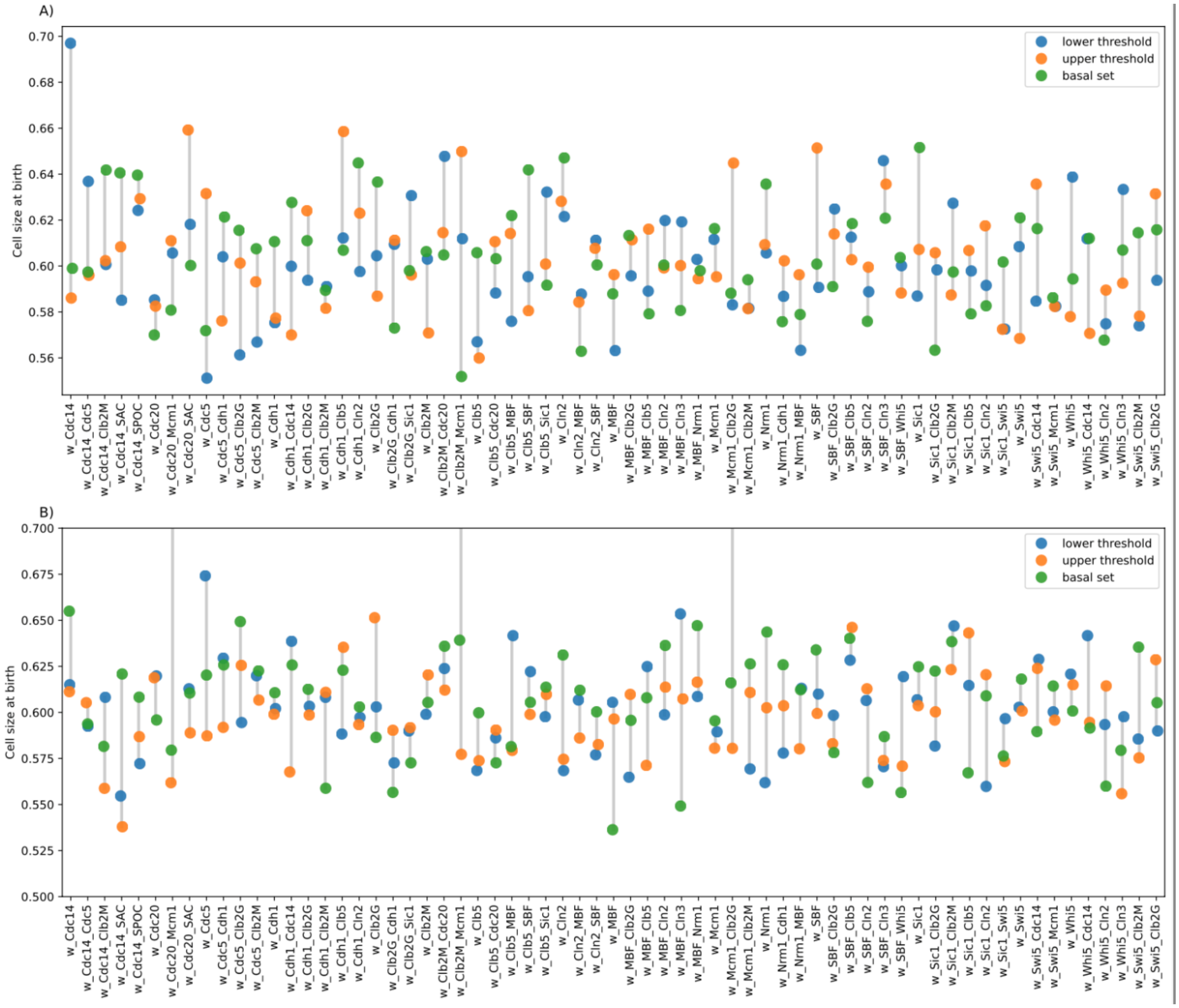

Supplementary Figure 2. Robustness of wild-type cell viability with respect to perturbations of the 65 ω_{ij} coefficients from the values given in Suppl. Table S2. Each parameter value is perturbed separately by a fixed percentage, and the average size of a newborn cell is calculated over the course of 10,000 updates of the Boolean model. (A) Perturb by ±40%. All simulated cycles are viable. (B) Perturb by ±50%. The three perturbations that cause cell cycle arrest are reductions: $\omega_{Cdc20,Mcm1}\longrightarrow 0.5$, $\omega_{Clb2M,Mcm1}\longrightarrow 0.5$, $\omega_{Mcm1,Clb2G}\longrightarrow 0.5$.

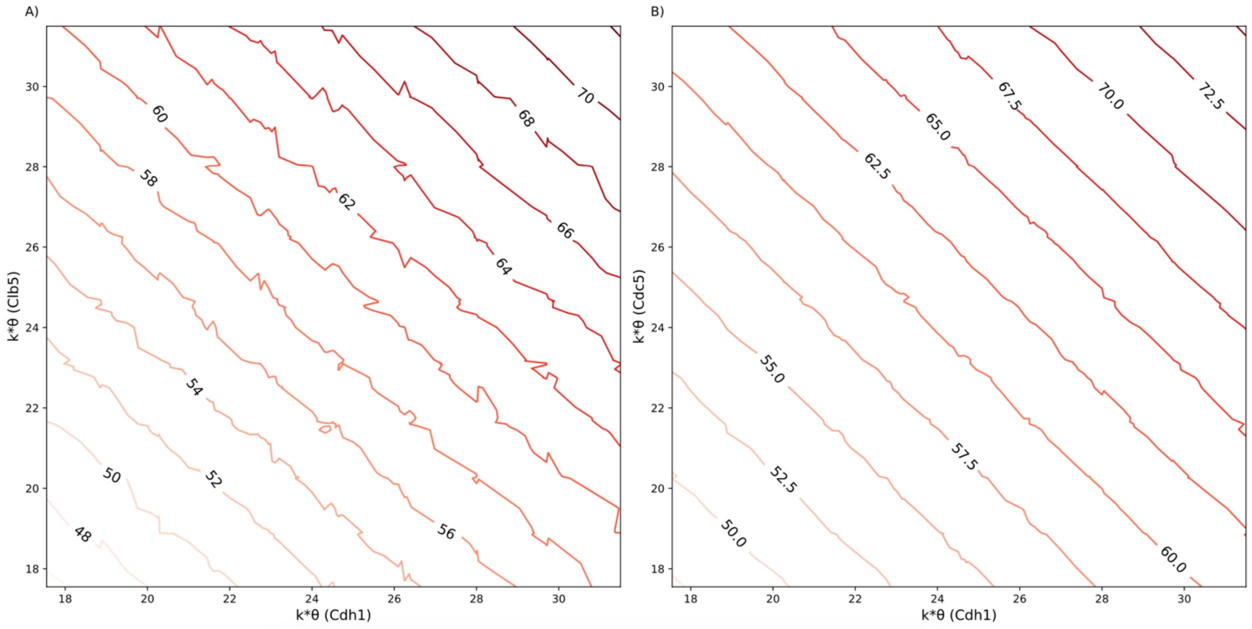

Supplementary Figure 3. Endocycle period depends linearly on the mean delays ($\Delta t_{\text{mean}} = k\cdot\theta$) of the two slow steps in the negative feedback loop. (A) Endoreplication period in dependence on delays of Cdh1 inactivation and Clb6 activation. (B) Cdc14 endocycle period in dependence on delays of Cdh1 activation and Cdc5 inactivation.

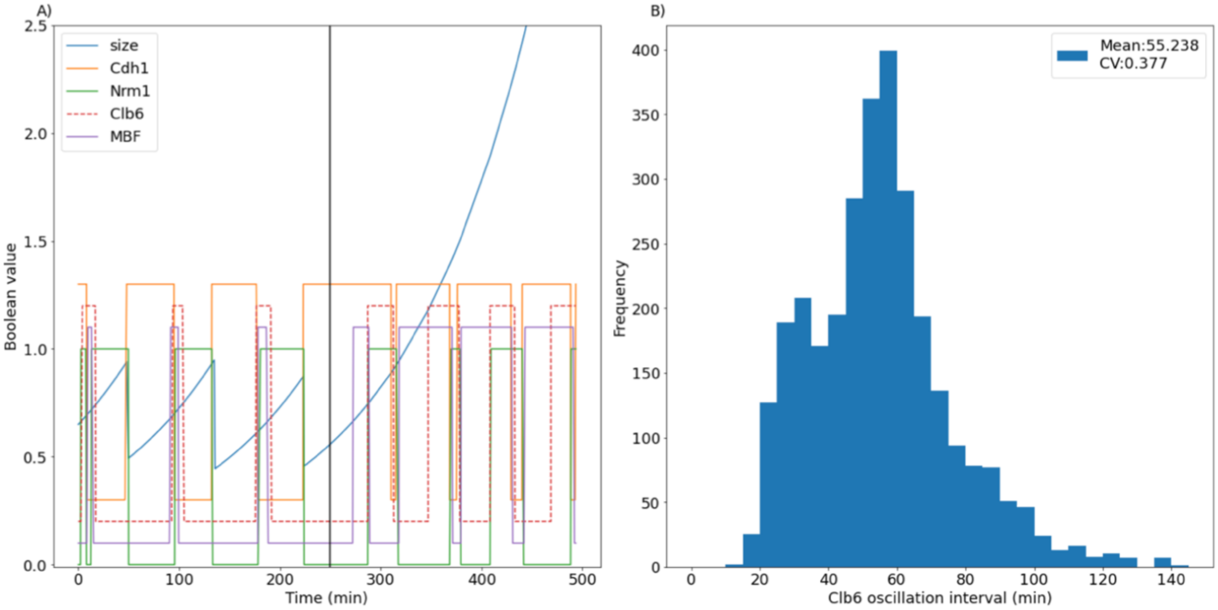

Supplementary Figure 4. Simulation of endoreplication cycles in the *clb1-5*Δ mutant strain with $\omega_{\mathbf{Nrm1}}$ = 1.5. (A) Wildtype cell cycles for t < 250 min, and endoreplication cycles for t > 250 min. *Cdh1*, *Clb6* and *MBF* are offset by 0.30, 0.20, and 0.10, respectively. (B) The bimodal distribution of Clb6 oscillation periods.

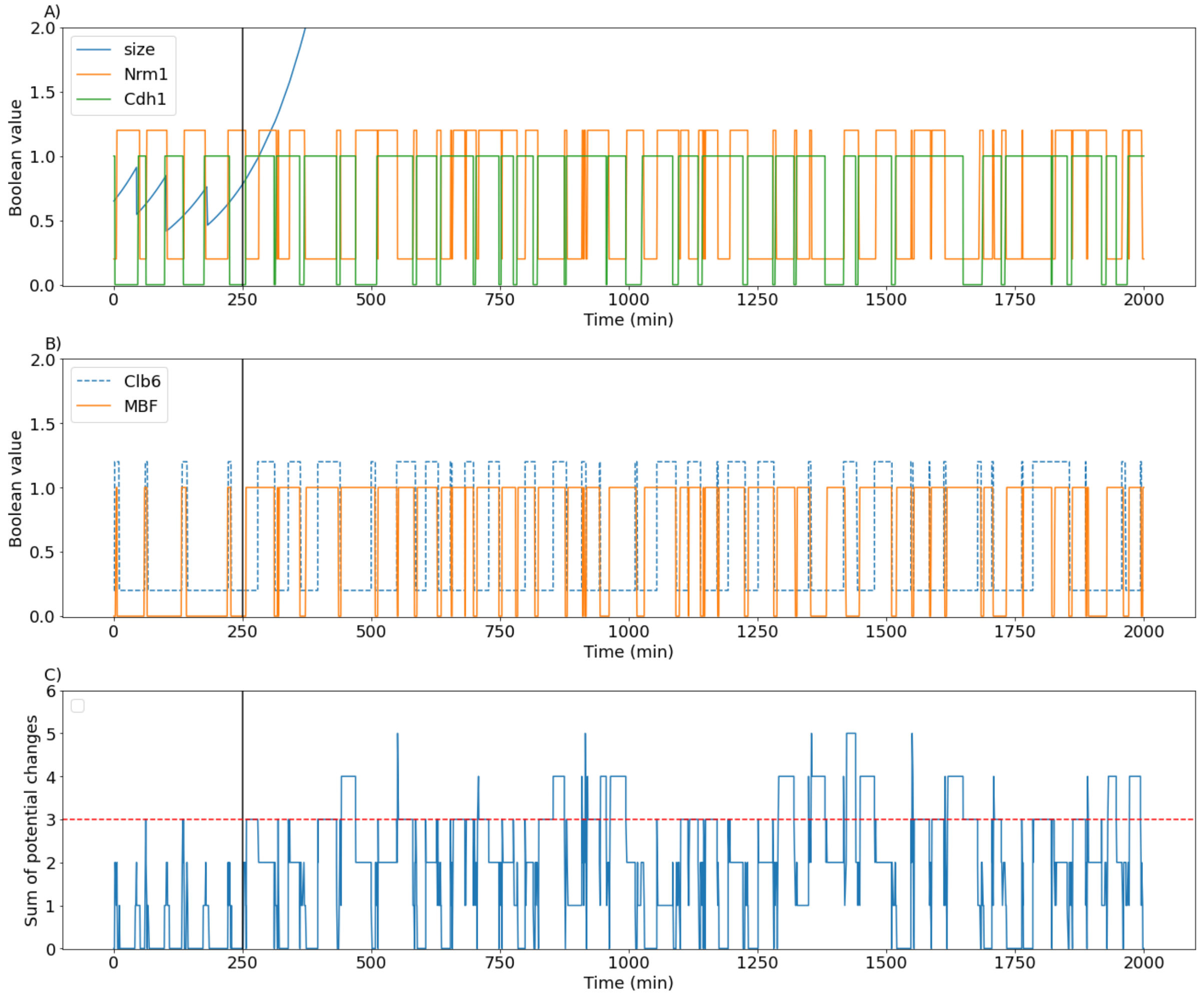

Supplementary Figure 5. Simulation of endoreplication cycles in the *clb1-5*Δ mutant strain with $\omega_{\mathbf{Nrm1}}$ = 1.5. (A and B) The four proteins that drive endoreplication cycles. Clb6 and Nrm1 are offset by 0.2. (C) The sum of the potential changes of five proteins (Nrm1, Clb6, Cln2, Cdh1, and Sic1).

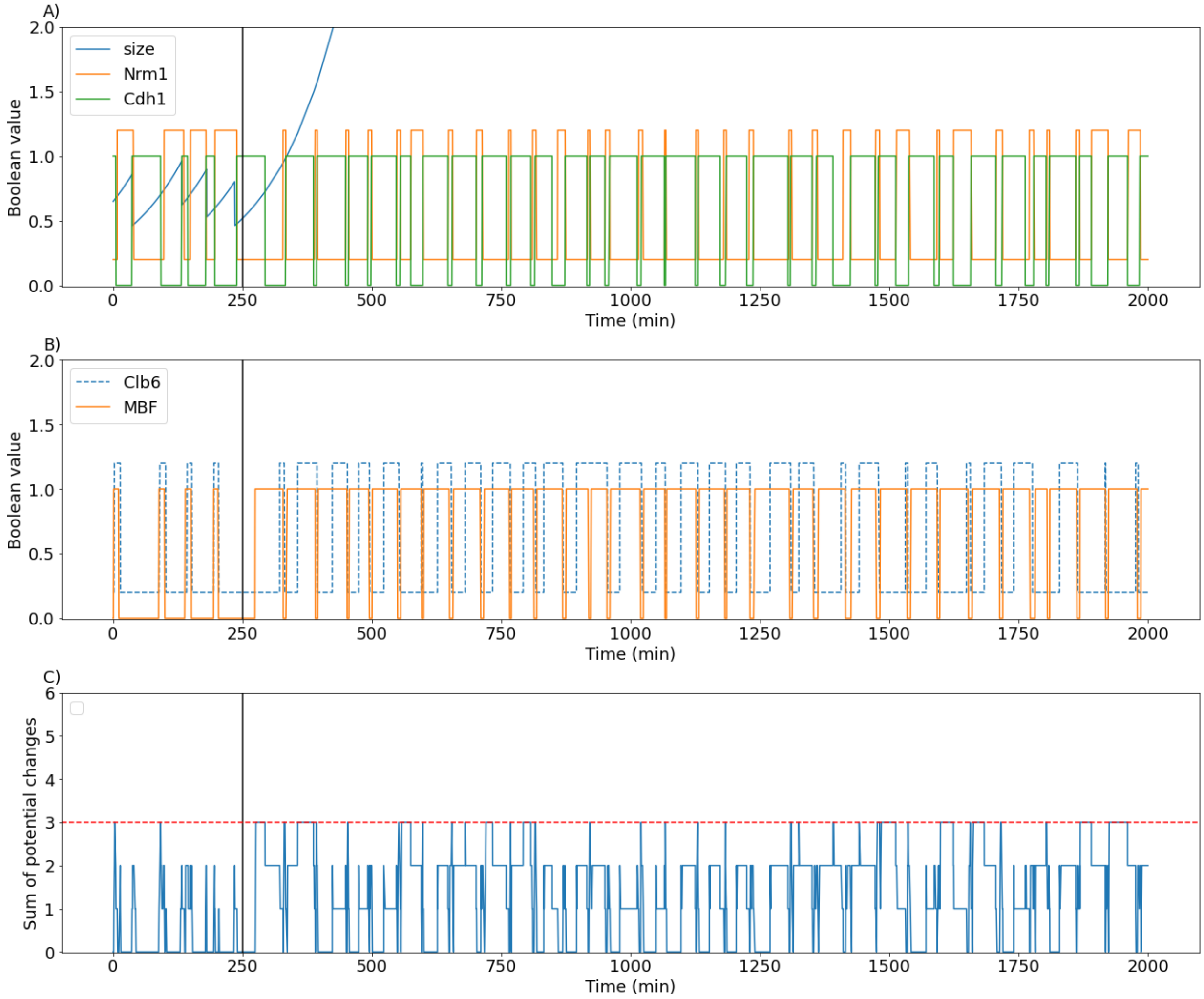

Supplementary Figure 6. Simulation of endoreplication cycles in the *clb1-5*Δ mutant strain with $\omega_{\mathbf{Nrm1}}$ = 0.5. (A and B) The four proteins that drive endoreplication cycles. Clb6 and Nrm1 are offset by 0.2. (C) The sum of the potential changes of five proteins (Nrm1, Clb6, Cln2, Cdh1, and Sic1).

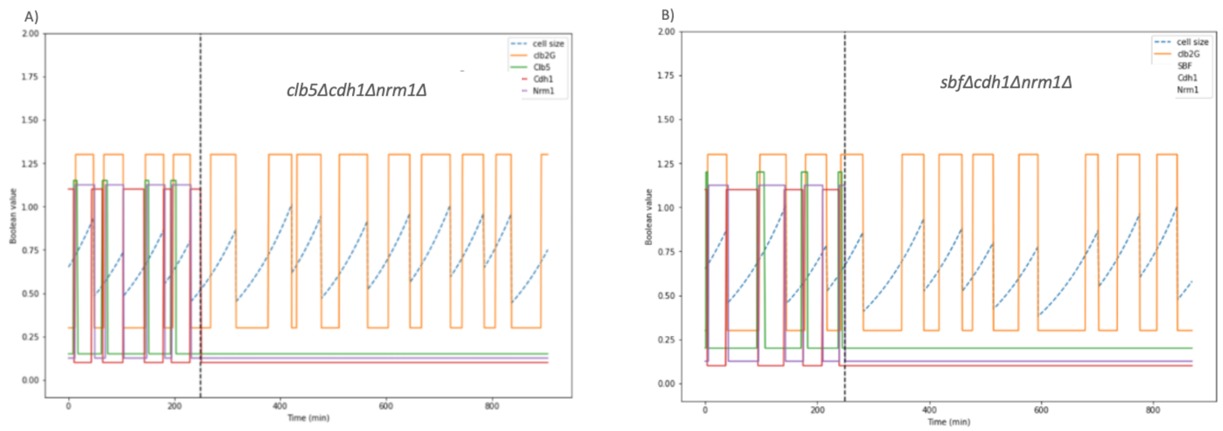

Supplementary Figure 7. Simulation of the triple mutants: (A) *cdh1Δ clb5Δ nrm1Δ* and (B) *cdh1Δ sbfΔ nrm1Δ.* The vertical dashed lines indicate the starting time of triple mutant simulation.

Supplementary Table 1. Model variables associated to molecular components of the budding yeast cell-cycle control system in Figure 1. CDK, cyclin-dependent kinase.

Variable	Regulatory Proteins	Functions
Whi5	Whi5	Inhibitor of SBF
SBF	Swi4:Swi6	Transcription factor for Cln2 and less for Clb5
MBF	Mbp1:Swi6	Transcription factor for Clb5 and less for Cln2
Nrm1	Nrm1	Inhibitor of MBF
Clb5	Clb5:Cdc28, Clb6:Cdc28	CDK that initiates DNA replication
Cln2	Cln2:Cdc28, Cln1:Cdc28	CDK that initiates bud emergence
Sic1	Sic1, Cdc6	Inhibitor of Clb5 and Clb2G
Cdh1	Cdh1:APC/C	Ubiquitin ligase, degradation of Nrm1 and Clb's in G_1
Clb2G	Clb2:Cdc28, Clb1:Cdc28	Clb2 activity in G_2 phase
Clb2M	Clb2:Cdc28, Clb1:Cdc28	Enhanced Clb2 activity in M phase
Mcm1	Mcm1	Transcription factor for Clb2
Cdc5	Cdc5	Polo-like kinase, activator of Cdc14
Cdc20	Cdc20:APC/C	Ubiquitin ligase, degradation of Clb's in anaphase
Cdc14	Cdc14	Phosphatase, activates Swi5 and Cdh1
Swi5	Swi5	Transcription factor for Sic1
**Variable**	**Checkpoint Proteins**	**Functions**
Cln3	Cln3:Cdc28, Bck2	CDK that inhibits Whi5 and activates SBF & MBF
SAC	Mad1:Mad2	Spindle assembly checkpoint (inhibits Cdc20)
SPOC	Lte1, Cdc15:Tem1	Spindle position checkpoint (inhibits Cdc14)
**Variable**	**Progression**	**Functions**
Size	Cell growth	Inhibits Cln3 until size > S_0
SPN	Spindle assembly progress	Inhibits SAC until SPN > 1
SPO	Spindle position progress	Inhibits SPOC until SPO > 1
**Flag**	**Proteins**	**Function**
ORI	Mcm2-7, Cdc6	Origin licensing factor (0 = licensed, 1 = fired)

Supplementary Table 2. Model parameter values.

A. Parameters defining the Boolean functions			
Parameter	Value	Parameter	Value
ω_{Cdc14}	−0.6	$\omega_{\mathrm{MBF,Clb2G}}$	4
$\omega_{\mathrm{Cdc14,Cdc5}}$	1.5	$\omega_{\mathrm{MBF,Clb5}}$	3
$\omega_{\mathrm{Cdc14,Clb2M}}$	1	$\omega_{\mathrm{MBF,Cln2}}$	2
$\omega_{\mathrm{Cdc14,SAC}}$	1	$\omega_{\mathrm{MBF,Cln3}}$	1
$\omega_{\mathrm{Cdc14,SPOC}}$	1	$\omega_{\mathrm{MBF,Nrm1}}$	5
ω_{Cdc20}	−0.5	ω_{Mcm1}	−0.5
$\omega_{\mathrm{Cdc20,Mcm1}}$	1	$\omega_{\mathrm{Mcm1,Clb2G}}$	1
$\omega_{\mathrm{Cdc20,SAC}}$	1	$\omega_{\mathrm{Mcm1,Clb2M}}$	3
ω_{Cdc5}	−0.5	ω_{Nrm1}	0.5
$\omega_{\mathrm{Cdc5,Cdh1}}$	3	$\omega_{\mathrm{Nrm1,Cdh1}}$	2
$\omega_{\mathrm{Cdc5,Clb2G}}$	3	$\omega_{\mathrm{Nrm1,MBF}}$	1
$\omega_{\mathrm{Cdc5,Clb2M}}$	3	ω_{SBF}	−0.5
ω_{Cdh1}	0.5	$\omega_{\mathrm{SBF,Clb2G}}$	4
$\omega_{\mathrm{Cdh1,Cdc14}}$	3	$\omega_{\mathrm{SBF,Clb5}}$	1
$\omega_{\mathrm{Cdh1,Clb2G}}$	1	$\omega_{\mathrm{SBF,Cln2}}$	1
$\omega_{\mathrm{Cdh1,Clb2M}}$	3	$\omega_{\mathrm{SBF,Cln3}}$	1
$\omega_{\mathrm{Cdh1,Clb5}}$	1	$\omega_{\mathrm{SBF,Whi5}}$	3
$\omega_{\mathrm{Cdh1,Cln2}}$	0.6	ω_{Sic1}	1.5
ω_{Clb2G}	0.5	$\omega_{\mathrm{Sic1,Clb2G}}$	3
$\omega_{\mathrm{Clb2G,Cdh1}}$	1	$\omega_{\mathrm{Sic1,Clb2M}}$	3
$\omega_{\mathrm{Clb2G,Sic1}}$	1	$\omega_{\mathrm{Sic1,Clb5}}$	2
ω_{Clb2M}	−0.5	$\omega_{\mathrm{Sic1,Cln2}}$	2
$\omega_{\mathrm{Clb2M,Cdc20}}$	2	$\omega_{\mathrm{Sic1,Swi5}}$	2
$\omega_{\mathrm{Clb2M,Mcm1}}$	1	ω_{Swi5}	−1.5
ω_{Clb5}	−0.5	$\omega_{\mathrm{Swi5,Cdc14}}$	2
$\omega_{\mathrm{Clb5,Cdc20}}$	0.2	$\omega_{\mathrm{Swi5,Mcm1}}$	2
$\omega_{\mathrm{Clb5,MBF}}$	3	$\omega_{\mathrm{Swi5,Clb2G}}$	1
$\omega_{\mathrm{Clb5,SBF}}$	0.8	$\omega_{\mathrm{Swi5,Clb2M}}$	1
$\omega_{\mathrm{Clb5,Sic1}}$	0.25	ω_{Whi5}	0.5
ω_{Cln2}	−0.9	$\omega_{\mathrm{Whi5,Cdc14}}$	1
$\omega_{\mathrm{Cln2,MBF}}$	0.5	$\omega_{\mathrm{Whi5,Cln2}}$	1
$\omega_{\mathrm{Cln2,SBF}}$	0.8	$\omega_{\mathrm{Whi5,Cln3}}$	1

ω_{MBF}	−0.5		
**B. Specific growth rate**			
r (glucose)	0.0077 min^{-1}	r (galactose)	0.0046 min^{-1}
**C. Gamma distribution**			
k	3	θ	0.3 min^{-1}
**D. Lognormal distributions**			
f_{mean} (glucose)	0.55	f_{CV} (glucose)	0.1
f_{mean} (galactose)	0.58	f_{CV} (galactose)	0.1
S_{0_mean}	0.60	S_{0_CV}	0.1
SPN_{mean}	0.07	SPN_{CV}	0.03
SPO_{mean}	0.07	SPO_{CV}	0.03

Supplementary Table 3. The logical functions for the Boolean network used to update the 15 proteins of the cell-cycle control system.

1	Whi5 = (Cdc14 AND NOT(Cln2 AND Cln3)) OR (NOTCdc14 AND NOT(Cln2 OR Cln3))
2	SBF = (Cln3 OR Cln2 OR Clb5) AND NOT(Whi5 OR Clb2G)
3	MBF = (Clb5 AND Cln2 AND NOTNrm1) OR<br>(Clb5 AND NOTClb2G AND NOTNrm1) OR<br>(Cln2 AND NOTClb2G AND NOTNrm1) OR<br>(Cln3 AND NOTClb2G AND NOTNrm1) OR<br>(Clb5 AND Cln2 AND Cln3 AND NOTClb2G)
4	Nrm1 = NOTCdh1
5	Clb5 = MBF OR (SBF AND NOTCdc20) OR (SBF AND NOTSic1)
6	Cln2 = SBF AND MBF
7	SIC1 = (Swi5 AND NOTClb2G AND NOTClb2M AND NOTClb5) OR<br>(Swi5 AND NOTClb2G AND NOTClb2M AND NOTCln2) OR<br>(Swi5 AND NOTClb2G AND NOTClb5 AND NOTCln2) OR<br>(Swi5 AND NOTClb2M AND NOTClb5 AND NOTCln2) OR<br>(NOTClb2G AND NOTClb2M AND NOTClb5 AND NOTCln2)
8	Cdh1 = (Cdc14 AND NOTClb2M) OR<br>(Cdc14 AND NOTClb2G AND NOTClb5 AND NOTCln2) OR<br>(NOTClb2G AND NOTClb2M AND NOTClb5 AND NOTCln2)
9	Clb2G = NOT(Cdh1 OR Sic1)
10	Clb2M = Mcm1 AND NOTCdc20
11	Mcm1 = Clb2G OR Clb2M
12	Cdc5 = (NOTCdh1 AND (Clb2G OR Clb2M)) OR (Cdh1 AND Clb2G AND Clb2M)
13	Cdc20 = Mcm1 AND NOTSAC
14	Cdc14 = Cdc5 AND NOT(Clb2M OR SAC OR SPOC)
15	Swi5 = (Cdc14 AND Mcm1) OR (Cdc14 AND NOTClb2G AND NOTClb2M) OR (Mcm1 AND NOTClb2G AND NOTClb2M)

Supplementary Table 4. Mutant strains simulated by the model. * indicates inconsistency between simulated and observed phenotypes.

Item	Mutant	Modified Parameter (i any regulated proteins)	Phenotype		
			**Experiment (Reference)**	**ODE Model**	**Simulation**
1	WT in glucose	r = 0.0077	viable (size = 1.0) (PMID: 27187804)	viable (size = 1.0) (PMID: 27187804)	viable (size = 1.0)
2	WT in galactose	r = 0.0046 f_{mean} = 0.58	viable (size = 0.86) (PMID: 27187804)	viable (size = 0.73) (PMID: 27187804)	viable (size = 0.84)
3	*whi5Δ*	ω_{Whi5} = -10	viable (PMID: 12089449)	viable (PMID: 28725464)	viable
4	*sic1Δ*	ω_{Sic1} = -10	viable (PMID: 8614808)	viable (PMID: 15169868)	viable
5	*swi4Δ*	ω_{SBF} = -10	viable (PMID: 12024050)	viable (PMID: 28725464)	viable
6	*mbp1Δ*	ω_{MBF} = -10	viable (PMID: 8372350)	viable (PMID: 28725464)	viable
7	*cdc5Δ*	ω_{Cdc5}= -10	Inviable, no Cdc14 release (PMID: 11832211)	Inviable, no Cdc14 release (PMID: 28725464)	Inviable no Cdc14 release
8	*mcm1Δ*	ω_{Mcm1}= -10	Inviable (https://www.yeastgenome.org/locus/S000004646)	Inviable	Inviable
9	*cdc20Δ*	ω_{Cdc20}= -10	metaphase arrest (Cdc20 = 0 & Clb2 = 1) (PMID: 9501986)	Inviable (PMID: 15169868 & PMID: 28725464 & PMID: 27187804)	metaphase arrest (Cdc20 = 0 & Clb2G = Clb2M = 1)
10	*cdc14Δ*	ω_{Cdc14}= -10	Lethal-telophase arrest (cdc20 = 1 & clb2 = 0) (PMID: 9885559)	telophase arrest (cdc20 = 1 & clb2G = 1 & clb2M = 0) PMID: 15169868	telophase arrest (Cdc20 = 1 & Clb2G = 1 & Clb2M = 0)
11	*swi5Δ*	ω_{Swi5}= -10	viable (PMID:12140549)	viable (PMID: 27187804)	viable
12	*cdh1Δ*	ω_{Cdh1}= -10	viable but smaller than wild type (PMID: 9288748)	viable (PMID: 27187804 & PMID: 15169868)	viable
13	*nrm1Δ*	ω_{Nrm1}= -10	viable (PMID:12489128)	Not included	viable
14	WT in Nocodazole	SAC = 1	metaphase arrest (PMID: 10329618)	metaphase arrest (PMID: 27187804)	metaphase arrest (Cdc20 = 0 & Clb2G = Clb2M = 1)

15	*GAL-SIC1*	γ_{SIC1} = 0.4<br>*r* = 0.0046<br>f_{mean} = 0.58	viable<br>(PMID: 8164683)	viable<br>(PMID: 15169868)	viable
16	*GAL-CLN3*	γ_{CLN3}=0.5<br>*r* = 0.0046<br>f_{mean} = 0.58	viable<br>(PMID: 1316273)	viable<br>(PMID: 27187804)	viable
17	*GAL-CLB5*	γ_{CLB5} = 0.5<br>*r* = 0.0046<br>f_{mean} = 0.58	viable<br>(PMID: 8319908)	viable<br>(PMID: 15169868)	viable
18	*GAL-CDC5*	γ_{CDC5} = 0.5<br>*r* = 0.0046<br>f_{mean} = 0.58	viable<br>(PMID: 16455487)	viable<br>(PMID: 28725464)	viable
19	*GAL-CLB2*	γ_{CLB2}= 0.1<br>*r* = 0.0046<br>f_{mean} = 0.58	viable<br>(PMID: 27187804)	viable<br>(PMID: 27187804)	viable
20	*GAL-CDC20*	γ_{CDC20} = 0.5<br>*r* = 0.0046<br>f_{mean} = 0.58	Inviable<br>(PMID: 9482731)	inviable-mitotic catastrophe<br>(PMID: 28725464)	inviable-mitotic catastrophe (Cdc20 = 1 before SPN is finished)
21*	*GAL-CDC14*	γ_{CDC14} = 0.5<br>*r* = 0.0046<br>f_{mean} = 0.58	Inviable-G_1 arrest<br>(PMID: 27187804)	inviable-G_1 arrest<br>(PMID: 27187804)	Inviable-<br>S arrest
22	*cln1Δ cln2Δ*	ω_{Cln2} = -10	viable<br>(PMID: 7588610)	viable<br>(PMID: 28725464)	viable
23	*cln3Δ bck2Δ*	ω_{Cln3} = -10	inviable (G_1 arrest)<br>(PMID: 10545447)	inviable (G_1 arrest)<br>(PMID: 15169868 & PMID: 28725464)	inviable<br>(G_1 arrest: Sic1 = 1, ORI = 0)
24	*clb5Δ clb6Δ*	ω_{Clb5} = -10	viable (PMID: 8319908)	viable<br>(PMID: 28725464)	viable
25	*clb1Δ clb2Δ*	ω_{Clb2G} = -10<br>ω_{Clb2M} = -10	inviable (G_2 arrest)<br>(PMID: 1849457)	inviable (G_2 arrest)<br>(PMID: 28725464)	inviable (G_2 arrest)<br>(Clb2G = Clb2M = 0)
26*	*cln3Δ bck2Δ whi5Δ*	ω_{Cln3}= -10<br>ω_{Whi5} = -10	viable<br>(PMID: 15210110)	viable<br>(PMID: 28725464)	inviable
27	*cln3Δ bck2Δ sic1Δ*	ω_{Cln3}= -10<br>ω_{Sic1} = -10	inviable<br>(PMID: 10545447)	inviable (G_1 arrest)<br>(PMID: 28725464)	Inviable<br>(G_1 arrest: Cdh1 = 1, ORI = 0)
28	*cln1Δ cln2Δ cdh1Δ*	ω_{Cdh1}=-10<br>ω_{Cln2} = -10	viable<br>(PMID: 11809822)	viable<br>(PMID: 28725464)	viable
29	*cln1Δ cln2Δ clb5Δ clb6Δ*	ω_{Cln2} = -10<br>ω_{Clb5}= -10	inviable<br>(PMID: 7588610)	inviable (G_1 arrest)<br>(PMID: 15169868)	inviable (G_1 arrest)<br>(Sic1 = Cdh1 = 1)

30	*cln1Δ cln2Δ sic1Δ*	ω_{Cln2} = -10<br>ω_{Sic1} = -10	viable (PMID: 7588610)	viable (PMID: 15169868)	viable
31	*swi4Δ swi6Δ*	ω_{SBF} = -10<br>ω_{MBF} = -10	Inviable (PMID: 8372350)	Inviable (G_1 arrest) (PMID: 28725464)	Inviable
32	*swi5Δ cdh1Δ*	ω_{Swi5}= -10<br>ω_{Cdh1}= -10	Inviable (Arrest as binucleate cell) (PMID: 12960422)	Inviable (telophase arrest) (PMID: 15169868)	Inviable (telophase arrest) (Clb2G = 1 and ORI = 1)
33	*cdc20Δ clb5Δ clb6Δ*	ω_{Cdc20} = -10<br>ω_{Clb5} = -10	Inviable (Metaphase arrest) (PMID: 10647015)	Inviable (Metaphase arrest) (PMID: 15169868)	Metaphase arrest (Cdc20 = 0 & Clb2G = Clb2M = 1)
34	*sic1Δ cdh1Δ*	ω_{Cdh1}=-10<br>ω_{Sic1} = -10	Inviable (PMID: 9288748)	Inviable (PMID: 27187804)	inviable
35	*clb1Δ clb2Δ clb5Δ clb6Δ*	ω_{Clb5} = -10<br>ω_{Clb2G} = -10<br>ω_{Clb2M} = -10	Inviable (G_1 arrest) (PMID: 27187804)	Inviable (G_1 arrest) (PMID: 27187804)	Inviable (G_1 arrest: Sic1 = Cdh1 = 1)
36*	*GAL-SIC1 cln1Δ cln2Δ*	γ_{SIC1}= 0.4<br>ω_{Cln2} = -10<br>r = 0.0046<br>f_{mean} = 0.58	Inviable (PMID: 11809822)	Inviable G_1 arrest) (PMID: 15169868)	viable
37	*GAL-SIC1 cln1Δ cln2Δ cdh1Δ*	γ_{SIC1}= 0.4<br>ω_{Cln2}= -10<br>ω_{Cdh1}=-10<br>r = 0.0046<br>f_{mean} = 0.58	Inviable (PMID: 11809822)	Inviable (G_1 arrest) (PMID: 15169868)	inviable
38*	*GAL-SIC1 swi5Δ cdh1Δ*	γ_{SIC1}= 0.4<br>ω_{Cdh1} =-10<br>ω_{Swi5} =-10<br>r = 0.0046<br>f_{mean} = 0.58	viable (rescued) (PMID: 27187804)	viable (PMID: 15169868)	inviable
39*	*GAL-CLB5 sic1Δ*	γ_{CLB5} = 0.5<br>ω_{Sic1} = -10<br>r = 0.0046<br>f_{mean} = 0.58	Inviable (PMID: 27187804)	Inviable (ORI is not licensed) (PMID: 15169868)	viable (ORI is licensed)
40	*GAL-CLB5 cdh1Δ*	γ_{CLB5} = 0.5<br>ω_{Cdh1}=-10<br>r = 0.0046<br>f_{mean} = 0.58	Inviable for unknown cause (PMID: 27187804)	viable (PMID: 15169868)	inviable
41*	*GALL-CDC20 sic1Δ cdh1Δ*	γ_{CDC20} = 2<br>ω_{Cdh1}=-10<br>ω_{Sic1} = -10<br>r = 0.0046<br>f_{mean} = 0.58	viable (PMID: 27187804)	viable (PMID: 15169868)	inviable

42	*GAL-CLB2 cdh1Δ*	γ_{CLB2}= 0.1<br>ω_{Cdh1}=-10<br>r = 0.0046<br>f_{mean} = 0.58	Inviable (PMID: 27187804)	Inviable (telophase arrest) (PMID: 15169868)	inviable
43*	*GAL-CLB2 sic1Δ*	γ_{CLB2}= 0.1<br>ω_{Sic1} = -10<br>r = 0.0046<br>f_{mean} = 0.58	Inviable (telophase arrest) (PMID: 27187804)	Inviable (PMID: 15169868)	viable
44*	*GAL-CLB2 swi5Δ*	γ_{CLB2} = 0.1<br>ω_{Swi5} =-10<br>r = 0.0046<br>f_{mean} = 0.58	Inviable (PMID: 27187804)	Inviable (telophase arrest) (PMID: 15169868)	viable
45	*swi4Δ swi6Δ sic1Δ*	ω_{SBF} = -10<br>ω_{MBF} =-10<br>ω_{Sic1} = -10	Inviable (PMID: 10545447)	inviable (G_1 arrest) (PMID: 28725464)	inviable (G_1 arrest) (Cdh1 = 1)
46	*clb1-5Δ*	ω_{Clb2G}= -10<br>ω_{Clb2M}= -10<br>ω_{Clb5}= -1.1<br>$\omega_{i,\mathrm{Clb5}} \rightarrow$<br>$\omega_{i,\mathrm{Clb5}}/8$	clb6 oscillation (PMID: 22306294)	clb6 oscillation (PMID: 35504285)	Clb6 oscillation
47	*GAL-CLB2-dbΔ*	γ_{CLB2ND}= 0.5<br>r = 0.0046<br>f_{mean}=0.58	Cdc14 oscillation (PMID: 20660629)	Cdc14 oscillation (PMID: 28725464 & PMID: 35504285 & PMID: 27187804)	Cdc14 oscillation
48	*GAL-CLB2-dbΔ sic1Δ*	γ_{CLB2ND}= 0.5<br>ω_{Sic1} = -10<br>r = 0.0046<br>f_{mean} = 0.58	Cdc14 oscillation (PMID: 20660629)	Cdc14 oscillation (PMID: 28725464)	Cdc14 oscillation
49	*GAL-CLB2-dbΔ swi5Δ*	γ_{CLB2ND}= 0.5<br>ω_{Swi5}=-10<br>r = 0.0046<br>f_{mean} = 0.58	Cdc14 oscillation (PMID: 20660629)	Cdc14 oscillation (PMID: 28725464)	Cdc14 oscillation
50	*GAL-CLB2-dbΔ cdc5Δ*	γ_{CLB2ND}= 0.5<br>ω_{Cdc5}=-10<br>r = 0.0046<br>f_{mean} = 0.58	No cdc14 oscillation (PMID: 20660629)	Telophase arrest, cdc14 is not released (PMID: 28725464)	Telophase arrest (Cdc14 = 0)

Supplementary Table 5. Summary of time-delay parameters in endoreplication cycles and Cdc14 endocycles.

Protein with time delay	Description	Gamma distribution (Mean ± CV)	
		endoreplication	Cdc14 endocycle
Cdc5	Delay for Cdc5 inactivation due to high Clb2G&M from GAL promotor	0.9 ± 0.58	22.5 ± 0.25
Cdh1	Delay for Cdh1 activation due to high Clb2G&M level from GAL promotor	0.9 ± 0.57	22.5 ± 0.25
Cdh1	Delay for Cdh1 inactivation when Clb5 is deleted	22.5 ± 0.25	0.9 ± 0.58
Clb6	Delay for Clb6 activation because Clb6 has a lower transcription rate induced by MBF compared to Clb5	22.5 ± 0.25	0.9 ± 0.58

Supplementary Table 6. Comparison of four Boolean models of the budding-yeast cell-cycle engine. Using red and blue font indicates comparable elements of different models.

	Li et al. (2004)	Irons (2009)	Faure et al. (2009)<br>Core Model (Fig. 1)	Kittisak et al. (present work)
Components: Proteins	Cdh1, Sic1/Cdc6, Cln3/Bck2, SBF/MBF, Cln1/2, Clb5/6, Mcm1, Swi5, Clb1/2, Cdc20	Add: Yhp1/Yox1, Cdc14<br>FEAR<br>MEN	Add: Cdc14<br>Net1, Mad2, Pds1, Esp1<br>Cdc55, Bub2, Lte1, Cdc15	Add: Whi5, Nrm1, Cdc5, Clb2M, Cdc14<br>SAC<br>SPOC
Components: Events		S, B, M, CD	ORI, BUD, SPN, CYTOKINESIS	Size, ORI, SPN, SPO, CD
Boolean Functions	Heav(W_i(...))	Logical Functions<br>'Dummy' variables to delay some updates	Logical Functions<br>Clb2, Clb5, Cdc20 are multi-state variables	Heav(W_i(...))
Time	Discrete steps	Discrete steps	Discrete steps	Real variable
Cell Size	Absent	Absent	MASS (Boolean variable)	Size(t), real variable
Updating Method	Synchronous (deterministic)	Synchronous (deterministic)	Synchronous (deterministic)<br>Priorities for CYTOK & MASS	Asynchronous/random<br>Stochastic Δt & cell division
Cell Cycle	13 states from Start to a stationary, early G_1 state	19 state cycle, driven by NFL: Cln3 → SBF → Yhp1 --| Cln3	22 state cycle, activity of MASS in G_1 drives Start transition	Repetitive, noisy cycles driven by growth and CD
Mutants	Not simulated	Simulated 13 deletion strains and 4 checkpoints	Simulated 135 mutant strains by adjusting the logical functions in accordance with the genetics of each mutant strain	Simulated deletion and overproduction strains, endoreplication and Cdc14-endocycle strains; and predicted phenotypes of 105 'double-deletion' strains
Cell-cycle Distributions	Not applicable	Not applicable	Not applicable	Good fit to observed cell size and cycle-time distributions and to correlations between size at birth and G_1 duration
